# Integrative analyses of speciation and divergence in *Psammodromus hispanicus *(Squamata: Lacertidae)

**DOI:** 10.1186/1471-2148-11-347

**Published:** 2011-11-30

**Authors:** Patrick S Fitze, Virginia Gonzalez-Jimena, Luis M San-Jose, Diego San Mauro, Pedro Aragón, Teresa Suarez, Rafael Zardoya

**Affiliations:** 1 Department of Ecology and Evolution (DEE), Université de Lausanne, Biophore, Lausanne, CH-1015, Switzerland; 2 Department of Biodiversity and Evolutionary Biology, Museo Nacional de Ciencias Naturales (MNCN-CSIC), Calle José Gutierrez Abascal 2, Madrid, E-28006, Spain; 3 Instituto Pirenaico de Ecología (IPE-CSIC), Avenida Regimiento de Galicia s/n, Jaca, E-22700, Spain; 4 Fundación Araid, Edificio Pignatelli, Paseo Maria Agustin 36, Zaragoza, E-50004, Spain; 5 Department of Zoology, The Natural History Museum, Cromwell Road, London, SW7 5BD, UK; 6 Department of Cellular and Molecular Physiopathology, Centro de Investigaciones Biologicas (CSIC), Calle Ramiro de Maetzu 9, Madrid, E-28040, Spain

## Abstract

**Background:**

Genetic, phenotypic and ecological divergence within a lineage is the result of past and ongoing evolutionary processes, which lead ultimately to diversification and speciation. Integrative analyses allow linking diversification to geological, climatic, and ecological events, and thus disentangling the relative importance of different evolutionary drivers in generating and maintaining current species richness.

**Results:**

Here, we use phylogenetic, phenotypic, geographic, and environmental data to investigate diversification in the Spanish sand racer (*Psammodromus hispanicus*). Phylogenetic, molecular clock dating, and phenotypic analyses show that *P*. *hispanicus *consists of three lineages. One lineage from Western Spain diverged 8.3 (2.9-14.7) Mya from the ancestor of *Psammodromus hispanicus edwardsianus *and *P*. *hispanicus hispanicus *Central lineage. The latter diverged 4.8 (1.5-8.7) Mya. Molecular clock dating, together with population genetic analyses, indicate that the three lineages experienced northward range expansions from southern Iberian refugia during Pleistocene glacial periods. Ecological niche modelling shows that suitable habitat of the Western lineage and *P*. *h*. *edwardsianus *overlap over vast areas, but that a barrier may hinder dispersal and genetic mixing of populations of both lineages. *P*. *h*. *hispanicus *Central lineage inhabits an ecological niche that overlaps marginally with the other two lineages.

**Conclusions:**

Our results provide evidence for divergence in allopatry and niche conservatism between the Western lineage and the ancestor of *P*. *h*. *edwardsianus *and *P*. *h*. *hispanicus *Central lineage, whereas they suggest that niche divergence is involved in the origin of the latter two lineages. Both processes were temporally separated and may be responsible for the here documented genetic and phenotypic diversity of *P*. *hispanicus*. The temporal pattern is in line with those proposed for other animal lineages. It suggests that geographic isolation and vicariance played an important role in the early diversification of the group, and that lineage diversification was further amplified through ecological divergence.

## Background

Species diversity emerges from the combination of both past and ongoing evolutionary and ecological processes driving speciation [1-3]. However, it is challenging to determine the relative contributions of historical and ecological factors in causing genetic differentiation [4]. The traditional classification of modes of speciation (allopatric, peripatric, parapatric, and sympatric) within a spatial context [5,6] is currently revisited in the light of recent studies that integrate phylogenetic, ecological, and geographical data [3,7,8]. In the last decade, evolutionary biologists have focused on discerning the mechanisms leading to reproductive isolation, and the field has witnessed major advances in determining the relative contribution of historical geographic barriers to diversification thanks to the possibility of linking geological and phylogenetic data [9]. In contrast, the elucidation of the contribution to diversification of ecologically-based divergent selection due to environmental differences has been hindered until recently by the difficulty of simultaneously gathering genetic, phenotypic, and environmental data within the framework of a single study [3]. However, the recent accumulation of environmental data and the development of ecological niche modelling allow overcoming these limitations and provide the basis for an integrative approach that combines phylogenetic and biogeographic data in order to explain the origin and large-scale distribution patterns of biodiversity [9]. Integrative analyses provide new insights on the factors driving diversification and speciation, and allow disentangling the effects of environment from those of historical barriers [8-12]. In particular, it is possible to test explicitly whether diversity can evolve in allopatry and under similar ecological conditions (i.e. niche conservatism; [2]) or whether different ecological environments (i.e. niche divergence) that promote divergent natural selection are at the root of diversification [13,14].

Here, we integrate phylogenetic, phenotypic, geographic, and environmental data to investigate the contributions of historical geographic barriers and environmental differences to speciation and divergence in the Spanish sand racer (*Psammodromus hispanicus*). The current distribution of *P*. *hispanicus *includes the Iberian Peninsula, and the French Mediterranean coast with an upper altitudinal limit at 1700 m a.s.l. [15]. The broad distribution of this group, which inhabits regions with very distinct habitats as well as areas with complex geological histories, makes it a suitable model to investigate how vicariant events due to geographical barriers and niche divergence due to selection have influenced diversification. *P*. *hispanicus *Fitzinger, 1826 consists of two subspecies, namely *P*. *hispanicus hispanicus *Fitzinger, 1826 and *P*. *hispanicus edwardsianus *(Dugès, 1829). The Iberian Peninsula hosts a second species of the same genus, *P*. *algirus *(Linnaeus, 1758), consisting of two divergent Eastern (E) and Western lineages, the latter including African, as well as Northwestern (NW) and Southwestern (SW) Iberian clades [16]. Other species of the genus *Psammodromus *are *P*. *blanci *(Lataste, 1880) from Algeria, Morocco, and Tunisia, and *P*. *microdactylus *(Boettger, 1881) endemic to Morocco.

Based on a representative sampling of *P*. *hispanicus *in Spain, we first reconstructed phylogenetic relationships among sampled populations using both mitochondrial (mt) and nuclear markers, and dated major cladogenetic events, comparing patterns observed in *P*. *hispanicus *with those of *P*. *algirus*. Second, we investigated differences between molecular lineages in phenotypic traits using multivariate analyses. Third, we performed ecological niche modelling and applied different procedures to assess niche divergence and the spatial structure of shared environmental conditions among lineages, in order to investigated the evolutionary and ecological processes that promoted genetic and phenotypic differentiation in the Spanish sand racer. More specifically, we tested whether niche divergence and/or allopatric speciation may explain the observed diversity. Under the niche divergence hypothesis we predicted that two closely related taxonomic groups would live in habitats characterized by different environmental conditions. Under the allopatric speciation hypothesis we predicted genetic, but not necessarily ecological divergence, and thus that environmental niches of sister species should be more similar than under ecological speciation. Finally, we speculate which geological events may have led to the observed diversity.

## Results

### Phylogenetic Relationships within *Psammodromus*

**Figure 1:**
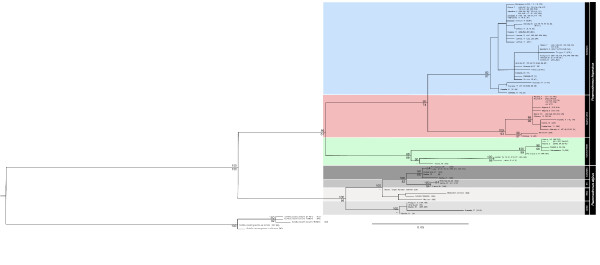
**Maximum likelihood phylogeny based of the mt data set including all 285 *Psammodromus *specimens**. The number above each branch refers to the Bayesian posterior probability (shown as percentage) of the node. Bootstrap values for ML are shown below branches. The sister group to *Psammodromus *(*Gallotia*) was used as an outgroup. The sample location and the population number are given. The specimen reference numbers are provided in brackets. For *P*. *algirus*, lineage names are given.

**Figure 2:**
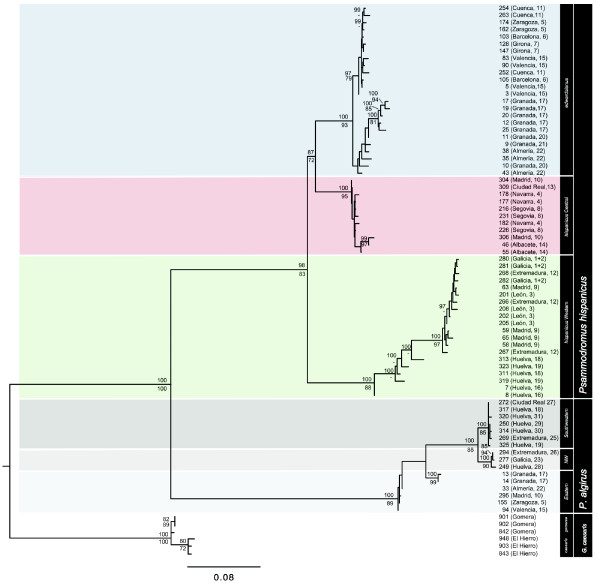
**Maximum likelihood phylogeny of the genus *Psammodromus *based on the combined data set**. The number above each branch refers to the Bayesian posterior probability (shown as percentage) of the node. Bootstrap values for ML are shown below branches. The sister group to *Psammodromus *(*Gallotia*) was used as an outgroup. The species, subspecies, and lineage name, and the specimen's reference number are given. The sample location and the population number are indicated in brackets.

Phylogenetic analyses of the mitochondrial (mt) cytochrome *b *(*cytb*) data set were based on a 249 bp alignment with 89 variable positions and 82 parsimony-informative sites. The maximum likelihood (ML) reconstructed tree is shown in Figure 1. The Bayesian inference (BI) tree recovered identical internal nodes to the ML tree and differed only in the arrangement of terminal nodes, which did not receive strong statistical support in either analysis. Two nuclear loci (suppressor of SWI4 1 and clone 17) were included in the nuclear data set. Phylogenetic analyses of the nuclear data set were based on a 1,168 bp alignment with 135 variable positions, and 88 parsimony-informative sites. The ML and BI reconstructed trees were identical in topology to each other (and with respect to the mt trees) regarding internal nodes, and differed in the arrangement of terminal nodes (results not shown). Phylogenetic analyses of the combined data set, which included mt *cytb*, mt *nad4 *and the two nuclear loci, were based on a 2,014 bp alignment with 417 variable positions, and 354 parsimony-informative sites. The ML reconstructed tree is shown in Figure 2. The BI tree was identical in topology to the ML tree regarding internal nodes and differed only in the arrangement of terminal nodes, which did not receive strong statistical support in either analysis.

**Figure 3:**
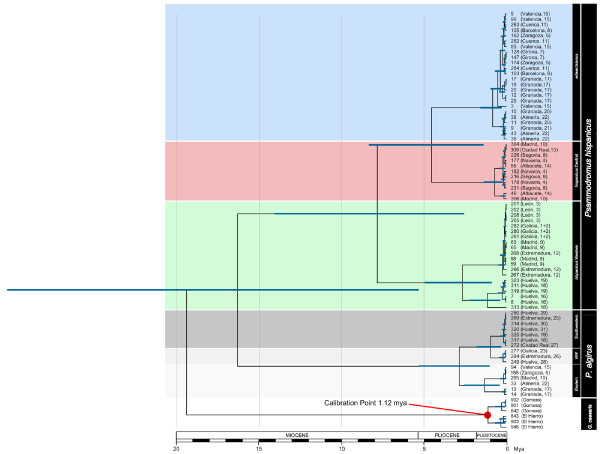
**Bayesian relaxed-clock timetree of the genus *Psammodromus *based on the combined data set**. The sister group to *Psammodromus *(*Gallotia*) was used as an outgroup and as calibration point for the molecular clock. The species, subspecies, and lineage name and the specimen's reference number are given. The sample location and the population number are indicated in brackets. Estimated ages and 95% confidence intervals are indicated.

**Figure 4:**
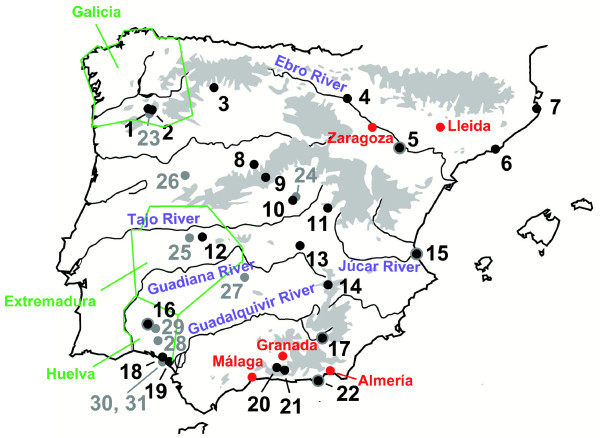
**Sampling locations and geographic localities mentioned in the article**. Numbers correspond to the population numbers indicated in Additional File 1 - Table S1. Black dots indicate populations where individuals of *P*. *hispanicus *were sampled and grey dots where individuals of *P*. *algirus *were sampled (black and grey dots: populations where individuals of both groups were sampled). Geographic localities mentioned in the article are indicated. Districts are delimited in green, rivers in blue, mountain systems in grey, and cities in red.

All recovered trees (based on the mt, nuclear, and combined data sets) indicated that *P*. *hispanicus *and *P*. *algirus *form two sister clades. Bayesian relaxed clock dating using the combined dataset estimated the split at approximately 17.25 ± 0.36 Mya ± SE (Figure 3). The recovered trees support the split of *P*. *algirus *into at least two main clades, which separated around 3.01 ± 0.07 Mya (Figures 1, 2, and 3). One of the clades represented the Eastern lineage (Figures 1, 2, and 3). The other clades included *P*. *algirus *from Morocco, and the Southwestern (SW) and Northwestern (NW) clades. These latter two clades were identified based on a *NADH dehydrogenase subunit 4 *(*nad4*)-phylogenetic tree, which included our specimens and six specimens with homologous sequences available in GenBank that belonged either to the Northwestern (former name: *P*. *manuelae*) or the Southwestern (former name: *P*. *jeanneae*) clades [17,18]. Although there was good statistical support for the monophyly of NW and SW clades (Figures 1 and 2), that of the African clade was not strong (Figure 1). The NW and the SW clades diverged around 1.00 ± 0.02 Mya (Figure 3). The geographic distribution of the NW and SW clades ranged from Huelva to Galicia and from Huelva to Extremadura respectively, whereas the Eastern lineage ranged from Granada to Zaragoza (Eastern Spain; see Figure 4 for geographic locations).

### Phylogenetic Relationships and Phylogeography of *P*. *hispanicus*

**Figure 5:**
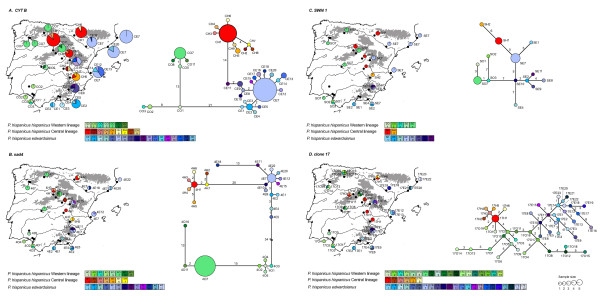
**Spatial distribution and diversity of mtDNA of *P. hispanicus***. Spatial distribution and diversity and Minimum-spanning networks of mt *cytb *(A), mt *nad4 *(B), nuclear suppressor of SWI4 1 (C) and nuclear clone 17 (D) sequence variation. Pie diagrams represent the haplotypes found at each sampling locality (black dots) and their relative abundance. The size of the pie is proportional to the sample size and the scale is identical for figure 5B, 5C and 5D, and differs for 5A. For minimum- spanning networks, each circle represents a haplotype and its size is proportional to its frequency in the population. Branches represent a single nucleotide change and numbers next to the branches correspond to the number of additional changes. Branch length is proportional to the number of changes.

*P*. *hispanicus *split into two well-supported lineages approximately 8.25 (2.9 - 14.7 CI) Mya (Figures 1, 2, and 3), one hereafter referred to as *P*. *hispanicus hispanicus *Western lineage (abbreviated as Western lineage), and the *P*. *hispanicus hispanicus *Central lineage (hereafter referred to as Central lineage) + *P*. *hispanicus edwardsianus *lineage (hereafter referred to as *edwardsianus *lineage). The split between the Central lineage and the *edwardsianus *lineage was dated 4.78 (1.5-8.7) Mya (Figures 1, 2, and 3). The monophyly of both the Central lineage and the *edwardsianus *lineage received strong statistical support in all phylogenetic analyses (Figures 1 and 2). The two mt and the two nuclear minimum-spanning networks support the existence of three independent lineages (Figure 5).

Within the Western lineage southern populations (Huelva) formed a paraphyletic assemblage with respect to northern populations. Within the *edwardsianus *lineage, southern populations (Granada and Almería) were recovered as the sister group of the northern populations (Figures 1 and 2). The southern-northern splits received strong statistical support in the combined analyses, and were further supported by statistically significant differences in the AMOVA (Table 1). While in the Western lineage and *edwardsianus *lineage most of the genetic variation was found among northern and southern groups, there was significant variation among populations and within groups in the *edwardsianus *lineage (Table 1). The main split within the Central lineage had no clear geographical correlation (all specimens recovered in the smaller clade belonged to populations where specimens belonging to the bigger clade were captured; Figures 1 and 2).

**Table 1:** Molecular differentiations in mtDNA between northern and southern groups of a) *P. hispanicus edwardsianus *and b) *P. hispanicus hispanicus *Western lineage.

	Variance	% total	*P*	Φ- statistic
*a) P*. *hispanicus edwardsianus*				
among groups	0.988	59.72	<0.0001	Φ_CT_= 0.597
among populations within groups	0.390	23.58	<0.0001	Φ_SC_= 0.585
within populations	0.276	16.70	0.004	Φ_ST_= 0.833
				
*b) P*. *hispanicus hispanicus *Western lineage				
among groups	9.023	96.76	<0.0001	Φ_CT_= 0.958
among populations within groups	0.093	0.99	0.018	Φ_SC_= 0.233
within populations	0.306	3.25	0.045	Φ_ST_= 0.968

The spatial distribution of mt *cytochrome b *(*cytb*) (Figure 5a), mt *nad4 *(Figure 5b), nuclear suppressor of SWI4 1 (Figure 5c) and nuclear clone 17 diversity (Figure 5d) showed current allopatry for the three main lineages recognized within *P*. *hispanicus*. There were statistically significant differences in the longitudinal distribution between the three lineages (*F*_2,18 _= 18.216, *P *< 0.001, all *post-hoc *contrasts were significant *P *< 0.05), but no differences in the latitudinal distribution (*F*_2,18 _= 0.519, *P *= 0.603). The *edwardsianus *lineage inhabits the eastern part of Spain (longitude: -1.153 ± 0.816 °, range: -3.7° - 3.2°), the Central lineage inhabits the central part (longitude: -3.057 ± 0.418 °, range: -4.3° - -2.0°), and the Western lineage the western part (longitude: -6.346 ± 0.394 °, range: -7.5° - -4.1°). In general, more different haplotypes and more haplotypes per geographic area were found in the southern populations compared to northern populations (Figure 5). The *cytb *haplotype (*h*) and nucleotide diversity (*π) *were higher in southern than in northern Spain (Table 2). *h *was significantly different in one and *π *in two lineages. The *cytb *minimum-spanning network indicated northward range expansion in all three main lineages (Figure 5b), while this pattern was less obvious in the nuclear network of the suppressor of SWI4 1 (Figure 5c). Tajima's *D *and Fu's *Fs *showed negative and statistically significant values for northern populations of all lineages, indicating population expansion, and non-significant values for southern populations in the Central lineage and the *edwardsianus *lineage (Table 2). Additionally, in the Western lineage Tajima's *D *was negative and statistically significant in southern populations suggesting population expansion. Overall all lineages, none of the two neutrality tests was significant (Table 2).

**Table 2:** Testing northward range expansion

		*π*	*h*	Tajima's *D*	Fu's *Fs*
Total		0.062	0.840	1.783	4.099
					
					
*edwardsianus *lineage		-1.213	6.363*		
	north	0.003	0.450	-1.505*	-4.830**
	south	0.013	0.786	0.624	-0.236
					
Central lineage		-1.611*	-3.284*		
	north	0.002	0.220	-2.269**	-3.372**
	south	0.011	0.667	0.072	0.502
					
Western lineage		0.042	5.288		
	north	0.002	0.246	-1.503*	-1.784*
	south	0.013	0.524	-1.623**	2.314

*P ≤ 0.05, **P ≤ 0.01

Tajima's *D *and Fu's *Fs *tests of selective neutrality on *cytb *data according to the three major lineages and northern and southern populations. Given is the nucleotide diversity (π), the haplotype diversity (*h*), Tajima's *D*, and Fu's *Fs*. Test statistics are based on 1000 simulations. Analyses were run for all individuals, for all lineages and for northern and southern populations of each lineage.

### Phenotypic Differences within *P*. *hispanicus*

**Figure 6:**
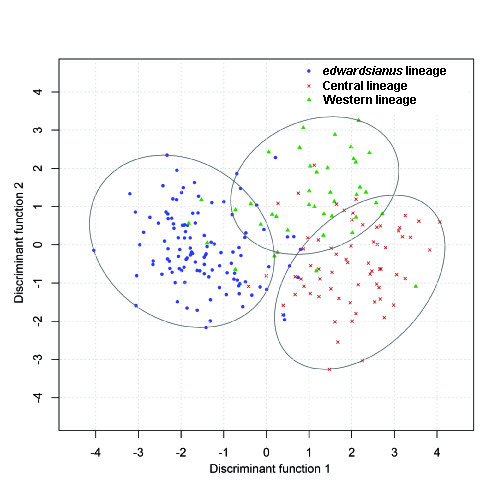
**Differences in phenotype among the three major lineages of *P. hispanicus***. The discriminant function scores derived from linear combinations of the phenotypic variables [88,89] are shown. Ellipses correspond to the clusters of the three lineages using the '*k-*means' clustering method [102]. Each cluster encloses the observations closest to lineage centroid. The three lineages are plotted using different colours: *P*. *hispanicus edwardsianus *blue, *P*. *hispanicus hispanicus *Central lineage red, *P*. *hispanicus hispanicus *Western lineage green.

Results from the permutational MANOVA (NP-MANOVA) showed that there were statistically significant differences in phenotypic traits between all three major lineages of *P*. *hispanicus *(*F*_2,208 _= 34.79, *P *< 0.001, Figure 6). Pairwise comparisons showed that each lineage differed from the other two lineages (*edwardsianus vs*. Central lineage: *t*_170 _= 7.75, *P_adj _<*0.001, *edwardsianus vs*. Western lineage: *t*_145 _= 5.29, *P_adj _<*0.001, Central *vs*. Western lineage: *t*_101 _= 3.38, *P_adj _= <*0.001).

Discriminant function analyses yielded two functions, the first explaining 88.14% of the variance and the second 11.86%. The first discriminant function separated *edwardsianus *from the other two lineages, whereas the second function discriminated between the Central and the Western lineage. Factor loadings (Table 3a) showed that the number of femoral pores, the nuptial coloration, and the number of throat scales were important determinants of the first discriminant function. The second discriminant function was mainly determined by the number of ocelli, the snout shape, and again by the nuptial coloration. The presence/absence of a supralabial scale below the subocular scale could not be included in the discriminant function analysis since no variance existed within lineages. In fact, all specimens belonging to the *edwardsianus *lineage showed a supralabial scale below the subocular scale whereas those from the other two lineages showed no scale below the subocular scale (χ^2 ^= 211, df = 2, *P *< 0.0001).

**Table 3:** Phenotypic differences between the three *P*. *hispanicus* lineages

	a. *Loadings*		**b**. ***Means ± SE per lineage***			**c**. ***Univariate ANOVAs***	
	
Variable	LF1	LF2	*edwardsianus *lineage	Central lineage	Western lineage	test statistic	*P*_adjusted_
Femoral pores (#)	-4.180	0.858	12.1 ± 0.1	9.9 ± 0.1	11.2 ± 0.2	*F *_2,208 _= 102.03	< 0.001
Throat scales (#)	-3.247	-0.165	20.4 ± 0.2	17.9 ± 0.2	18.9 ± 0.3	*F *_2,208 _= 40.55	< 0.001
Ocelli (#)	-2.387	1.473	1.9 ± 0.1	0.7 ± 0.1	2.0 ± 0.4	*F *_2,208 _= 31.71	< 0.001
SVL ratio	-2.262	-0.117	2.492 ± 0.038	2.199 ± 0.039	2.337 ± 0.053	*F *_2,208 _= 12.46	< 0.001
Snout shape	-0.524	1.221	1.065 ± 0.005	1.043 ± 0.006	1.098 ± 0.009	*F *_2,208 _= 12.36	< 0.001
Anal scale width (mm)	-0.304	0.446	0.063 ± 0.001	0.061 ± 0.001	0.066 ± 0.001	*F *_2,208 _= 3.77	0.024
Body mass (g)	1.192	-0.794	1.693 ± 0.03	1.877 ± 0.04	1.866 ± 0.07	*F *_2,208 _= 7.12	0.003
Ventral scales (#)	1.416	-0.741	24.6 ± 0.2	25.8 ± 0.3	25.1 ± 0.4	*F *_2,208 _= 6.55	0.004
Head ratio	1.423	-0.483	0.482 ± 0.003	0.500 ± 0.002	0.498 ± 0.004	*F *_2,208 _= 7.49	0.001
SVL (mm)	2.094	-0.882	46.39 ± 0.33	49.81 ± 0.43	48.31 ± 0.72	*F *_2,208 _= 18.04	0.001
Collar scales (#)	2.225	-1.185	0.3 ± 0.1	1.3 ± 0.2	0.7 ± 0.2	*H*^a^_2,208 _= 29.25	< 0.001
Nuptial coloration	3.774	1.266	0.5 ± 0.1	4.3 ± 0.3	5.2 ± 0.5	*H*^a^_2,208 _= 106.59	< 0.001

		**d.*****Post-hoc analyses***					
	
		Compared lineages					
	
**Variable**		*edwardsianus *vs Central		*edwardsianus *vs Western		Central vs Western	

Femoral pores (#)		*t*_170 _= 9.13, *P *< 0.0001		*t*_145 _= 5.09, *P *< 0.0001		*t*_101 _= 6.82, *P *< 0.0001	
Throat scales (#)		*t*_170 _= 9.03, *P *< 0.0001		*t*_145 _= 4.38, *P *= 0.0002		*t*_101 _= 2.92, *P *= 0.038	
Ocelli (#)		*t*_170 _= 7.60, *P *< 0.0001		*t*_145 _= 0.22, *P *= 0.830		*t*_101 _= 6.29, *P *< 0.0001	
SVL ratio		*t*_170 _= 5.20, *P *< 0.0001		*t*_145 _= 2.33, *P *= 0.105		*t*_101 _= 1.89, *P *= 0.366	
Snout shape		*t*_170 _= 2.53, *P *= 0.024		*t*_145 _= 3.28, *P *= 0.012		*t*_101 _= 5.13, *P *< 0.0001	
Anal scale width (mm)		*t*_170 _= 1.60, *P *= 0.111		*t*_145 _= 1.62, *P *= 0.429		*t*_101 _= 2.76, *P *= 0.055	
Body mass (g)		*t*_170 _= 3.51, *P *= 0.002		*t*_145 _= 2.46, *P *= 0.105		*t*_101 _= 0.47, *P *= 1.000	
Ventral scales (#)		*t*_170 _= 3.63, *P *= 0.002		*t*_145 _= 1.24, *P *= 0.537		*t*_101 _= 1.68, *P *= 0.483	
Head ratio		*t*_170 _= 3.29, *P *= 0.004		*t*_145 _= 3.01, *P *= 0.025		*t*_101 _= 0.21, *P *= 1.000	
SVL (mm)		*t*_170 _= 5.76, *P *< 0.0001		*t*_145 _= 2.43, *P *= 0.105		*t*_101 _= 1.53, *P *= 0.520	
Collar scales (#)		T^c^_BF _= 5.50, *P *< 0.0002		T^c^_BF _= 1.79, *P *= 0.537		T^b^_BF _= -2.44, *P *= 0.322	
Nuptial coloration		T^c^_BF _= 16.3, *P *< 0.0003		T^c^_BF _= 9.18, *P *< 0.0001		T^b^_BF _= 1.82, *P *= 0.537	

^a^Kruskal Wallis test; ^b^Behrens Fisher test

**a**. Factor loadings of each linear function (LF) derived from discriminant function analyses. **b**. Means (± SE) per lineage of the measured traits used for the phenotypic analyses. **c**. Results of univariate ANOVAs. **d**. *Post-hoc *tests allow understanding what lineages differ in what traits. All *P-*values are adjusted for multiple testing using Bonferroni procedures.

Results from univariate ANOVAs are shown in Table 3b and 3c. In brief, there were statistically significant differences between all three major lineages in the number of femoral pores, in the number of throat scales and in the snout shape. The number of ocelli differed between the Central lineage and the other two lineages, but no differences were present between *edwardsianus *and the Western lineage. The snout-to-vent length (SVL), SVL ratio, body mass, the number of ventral scales, and the number of collar scales differed between *edwardsianus *and the Central lineage, and there were no differences between the Western lineage and the two other lineages. There were statistically significant differences between the *edwardsianus *lineage and the other two lineages in head ratio and nuptial coloration, and no differences between Central and Western lineage.

### Ecological Niche Modelling within *P*. *hispanicus*

Five out of eight sampled populations (62.5%) that were previously unknown to the authors were located in 10 × 10 km squares where *P*. *hispanicus *has not been recorded previously [15]. This indicates that the distribution of *P*. *hispanicus *is underestimated, and thus biogeographic modelling may be importantly hindered when using presence/absence data. In this regard, the Spanish Atlas of Amphibians and Reptiles [15] does not discriminate between presences belonging to the different lineages. Consequently, we used modelling techniques that do not require absence data to link our presence records with environmental predictors and run Ecological Niche Factor Analysis (ENFA) based on a systematic population sampling (1 population per intersection of a ± 250 km grid) that included 22 populations (Figure 4, Additional File 1: Table S1).

#### Model Evaluation

We evaluated the model error of the niche models using an independent data-set obtained from the Official Spanish Atlas of Amphibians and Reptiles [15]. The model omission error was 13.7% with respect to the currently known presences of *P*. *hispanicus*, which is reasonably small. We further determined the importance of the sampling points for the lineage predictions using jackknife methodology, in order to investigate whether sampling points of a given lineage may be more important than in other lineages and thus whether differing sample sizes among lineages may have biased our predictions and conclusions. The average proportional habitat suitability (HS) difference was similar among lineages (one-way ANOVA: *F*_2,19 _= 0.91, *P *= 0.42), indicating similar population representativeness among lineages and no bias due to unequal sample sizes. These are important conditions for comparing niche divergences/similarities and overlaps among pair combinations of lineages.

#### Assessment of predictor relevance

**Figure 7:**
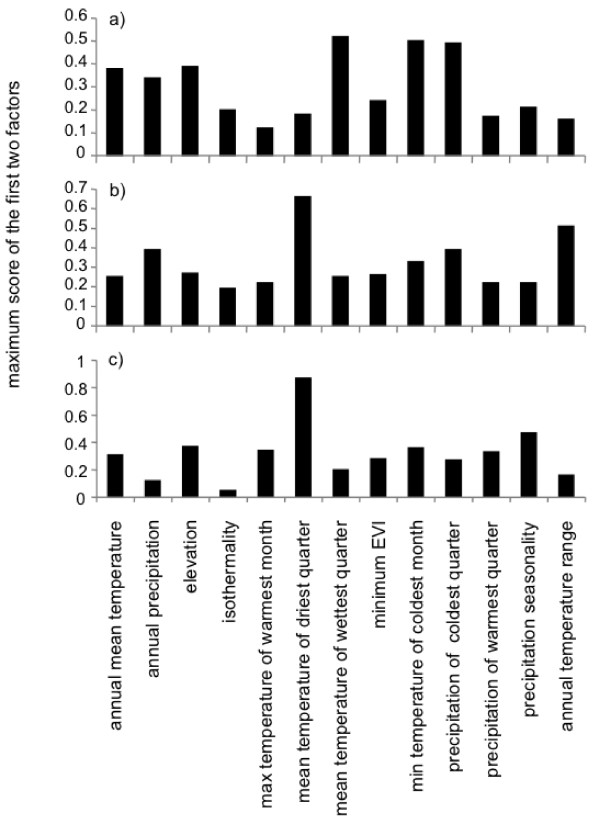
**Relative importance of thirteen ecogeographical variables for predicting lineage distribution using ENFA**. The absolute maximum coefficient value of the two most important ENFA factors is given for each predictor and each lineage model: a) *P*. *hispanicus edwardsianus*, b) *P*. *hispanicus hispanicus *Central lineage, c) *P*. *hispanicus hispanicus *Western lineage.

Most of the variation explained by ecological niche factor analysis (ENFA) was included in the two first factors (*edwardsianus *lineage: 97%; Central lineage: 89%; Western lineage: 96%). ENFA showed that the distribution of the *edwardsianus *lineage was best predicted by the mean temperature of wettest quarter, minimum temperature of the coldest month, and precipitation of the coldest quarter (Figure 7a). Mean temperature of the driest quarter and annual temperature range best predicted the distribution of the Central lineage, whereas the mean temperature of the driest quarter best predicted that of the Western lineage (Figures 7b and 7c).

Univariate ANOVAs of environmental predictors showed significant differences between lineages in environmental parameters (Table 4). The *edwardsianus *lineage inhabited habitats with higher mean temperature of the wettest quarter and higher minimum temperatures during the coldest month than the Central lineage. Both, the precipitation of the coldest quarter and Minimum Enhanced Vegetation Indexes (EVI) were significantly smaller than for the Western lineage, but did not differ from the populations of the Central lineage. The ecological niche of the Central lineage was characterized by lower minimum temperature of coldest month than the *edwardsianus *lineage (and a tendency compared to the Western lineage), lower precipitation of coldest quarter than the Western lineage (no differences compared to the *edwardsianus *lineage), lower precipitation seasonality than the other two lineages and lower minimum EVI than the Western lineage. The Western lineage occurs in habitats with lower mean temperature of wettest quarter than the *edwardsianus *lineage, higher precipitation of coldest quarter and higher minimum EVI than the other two lineages, and higher precipitation seasonality than the Central lineage (Table 4).

**Table 4:** Differences between the three lineages in environmental population parameters

Parameters	Test statistic		Contrasts (*P*)			Estimates (mean ± SE)			
	
	*F*_2,19_	*P*	*edwardsi* *anus*- Central	*edward* *sianus*- Western	Central- Western	Intercept	*edwardsianus* lineage	Central lineage	Western lineage
*Temperature parameters*									
Annual mean temperature	2.273	0.130				5810.1 ± 297.3	662.6 ± 394.9	-928.9 ± 458.2	266.4 ± 405.4
Max temperature of warmest month	0.043	0.958				3256.5 ± 78.5	9.956 ± 104.246	21.378 ± 120.969	-31.3 ± 107.0
Mean temperature of driest quarter	0.292	0.750				4945.4 ± 327.7	-26.469 ± 438.302	-213.645 ± 505.132	240.113 ± 446.93
Mean temperature of wettest quarter	3.546	0.049	0.177	0.016	0.391	168.728 ± 7.016	22.606 ± 9.319	-4.128 ± 10.814	-18.478 ± 9.568
Min temperature of coldest month	2.779	0.087	0.036	0.759	0.067	136.538 ± 5.775	12.344 ± 7.671	-20.716 ± 8.902	8.372 ± 7.876
Annual temperature range	2.333	0.124				1156.6 ± 52.3	-88.0 ± 69.5	174.1 ± 80.7	-86.1 ± 71.4
Isothermality	0.610	0.554				258.175 ± 4.548	-5.177 ± 6.041	-0.716 ± 7.010	5.892 ± 6.203
*Precipitation parameters*									
Annual precipitation	2.520	0.107				5.901 ± 0.054	-0.112 ± 0.071	-0.038 ± 0.083	0.150 ± 0.073
Precipitation of coldest quarter	6.621	0.007	0.789	0.003	0.016	7.253 ± 0.147	-0.403 ± 0.195	-0.302 ± 0.226	0.705 ± 0.200
Precipitation of warmest quarter	0.446	0.647				7.155 ± 0.420	0.014 ± 0.558	0.499 ± 0.648	-0.513 ± 0.573
Precipitation seasonality	3.752	0.042	0.090	0.258	0.013	10.283 ± 0.343	0.223 ± 0.456	-1.329 ± 0.529	1.106 ± 0.468
*Vegetation parameters*									
Minimum EVI^a^	5.494	0.013	0.564	0.005	0.043	14.077 ± 0.062	-0.171 ± 0.082	-0.082 ± 0.096	0.252 ± 0.085
Elevation	2.091	0.151				70.177 ± 7.974	-15.156 ± 10.592	24.713 ± 12.291	-9.557 ± 10.875
									

^a^test statistics correspond to transformed variables, estimates correspond to the untransformed variable

Univariate ANOVAs testing for differences between the three major lineages of *P*. *hispanicus *and the 13 different box-cox transformed parameters measured at each sampled population and used for the ecological niche modelling.

#### Predictive maps of habitat suitability and suitability overlaps

**Figure 8:**
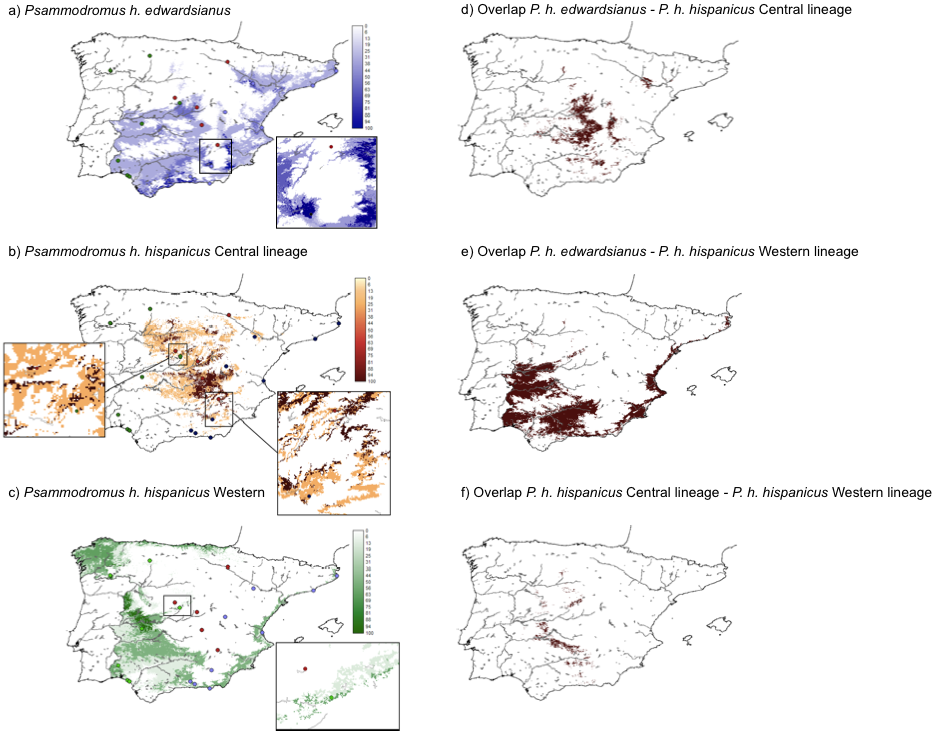
**Habitat suitability maps of *P. hispanicus *lineages**. Habitat suitability maps derived from ecological niche models for *P*. *hispanicus edwardsianus *(a), *P*. *hispanicus hispanicus *Central lineage (b) and *P*. *hispanicus hispanicus *Western lineage (c) using seven temperature, four precipitation, one vegetation and one topographic predictor. Sampled populations are indicated by circles (*P*. *hispanicus edwardsianus *populations in blue, *P*. *hispanicus hispanicus *Central lineage populations in red, Western lineage populations in green), and habitat suitability scores are listed in a graded colour series on the left border of the map. Potential contact zones (see Additional File 3 - Figure S3) are enlarged in separate panels. Overlapping habitat suitability predictions for *P*. *hispanicus edwardsianus *and *P*. *hispanicus hispanicus *Central lineage (d), *P*. *hispanicus hispanicus *Western lineage and *P*. *hispanicus edwardsianus *(e), and *P*. *hispanicus hispanicus *Central lineage and *P*. *hispanicus hispanicus *Western lineage (f) are given in separate panels. In these maps areas are coloured in brown if the habitat suitability scores of a lineage pair were larger than zero for both lineages.

For the *edwardsianus *lineage the highest habitat suitability (HS) scores were located in Eastern Spain (Figure 8a and Additional File 2: Figure S1a) ranging from the French border to the southernmost tip of Spain. Suitable habitat was located in the proximity of the East coast and also along the rivers flowing out into the Mediterranean Sea (e.g. Ebro River) and Atlantic Ocean (e.g. Guadalquivir River). Suitable habitat was also predicted in central Spain mainly close to the Tajo River and the Guadiana River. Suitable habitat for the Central lineage was predicted in central Spain (Figure 8b and Additional File 1: Figure S1b), and for the Western lineage in Western Spain and along the Mediterranean and Cantabrian coastline (Figure 8c and Additional File 1: Figure S1c). The zoomed potential contact zones (localized using minimum-convex polygons, Additional File 3: Figure S3) showed no connectivity between the *edwardsianus *and the Central lineage through HS values obtained from the *edwardsianus *model (Figure 8a, HS scores equal 100 and 0 respectively) and for the Central lineage suitability prediction (Figure 8b, HS scores equal 20 and 100 respectively). Similarly, there was also a clear gap between the Central lineage and the Western lineage in both suitability predictions (Figures 8b and 8c; HS scores equal 0 and 50 for the Central lineage prediction and 100 and 0 for the Western lineage prediction). The maps of the overlapping habitat suitability showed overlap between the *edwardsianus *and Central lineage in central Spain and close to the Ebro River (Zaragoza-Lleida) (extent of overall overlap = 9.4%; Figure 8d, Additional File 2: Figure S2d, Additional File 2: Table S2), that the habitat suitability distributions of the *edwardsianus *and Western lineage overlapped over an important part of their distributions (extent of overlap = 23.4%; Figure 8e, Additional File 2: Table S2), and that almost no overlap existed between the Central and the Western lineage (2.8%; Figure 8f, Additional File 2: Table S2).

### Ecological Niche Divergence within *P*. *hispanicus*

**Figure 9:**
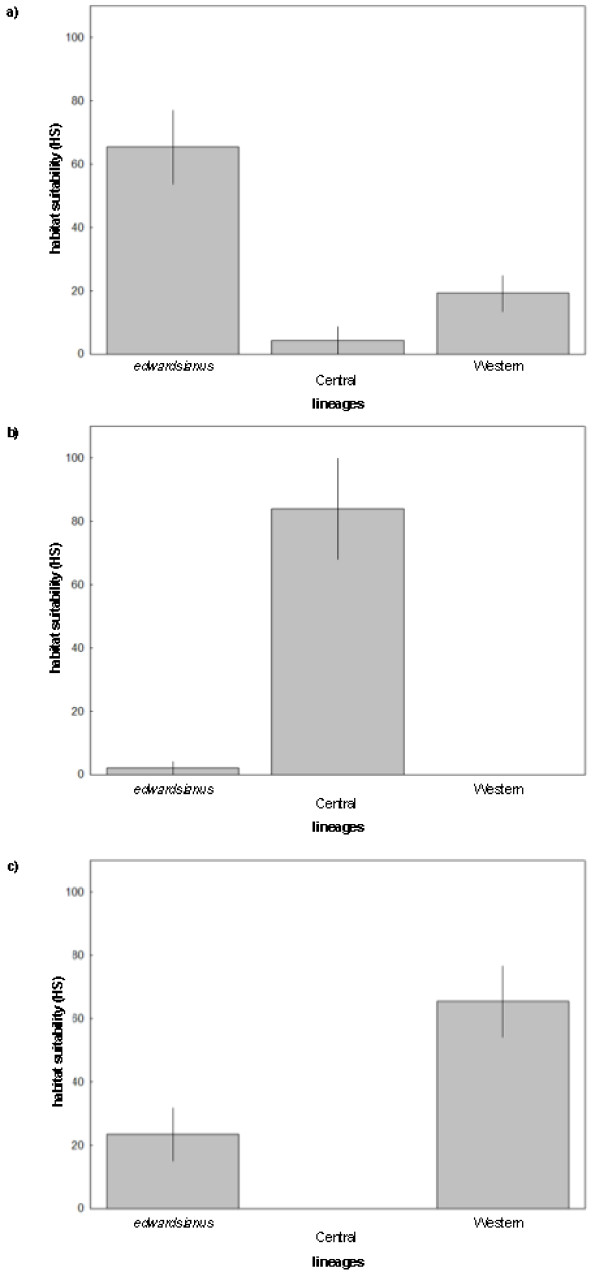
**Habitat suitability scores of the *P. hispanicus *lineages**. Differences in habitat suitability scores (HS) between *P*. *hispanicus *lineages, predicted by ENFA. Average (± SE) HS scores derived from models for the a) *edwardsianus *lineage, b) Central lineage, and c) Western lineage for the sampled populations are shown.

We first assessed niche divergence among lineages based on the principle of interpredictivity among lineages (see methods). There existed statistically significant differences between lineages in HS scores obtained for the sampled *edwardsianus *populations (*F*_2,19 _= 12.10, *P *< 0.001, Figure 9a and Additional File 2: Figure S2a). *Post-hoc *comparisons showed that the HS scores of sampled *edwardsianus *populations were higher than those of the other two lineages (Tukey range tests: *P_adj _*< 0.01 in both cases), and no significant differences were present between Central and the Western lineage populations (*P_adj _*= 0.45). Similarly, the HS for the Central lineage were significantly different between lineages (*F*_2,19 _= 54.84, *P *< 0.001, Figure 9b and Additional File 2: Figure *S2b)*. *Post-hoc *comparison showed that HS scores for the Central lineage populations were higher than for populations of the other two lineages (*P_adj _*< 0.001 in both cases) and again no statistically significant differences were present between the other two lineages (*P_adj _*= 0.90). HS scores derived for the Western lineage were also significantly different among lineages (*F*_2,19 _= 12.62, *P *< 0.001, Figure 9c and Additional File 2: Figure S2c), and *post- hoc *test showed that populations of the Western lineage had higher values than populations of the other two lineages (*P_adj _*< 0.05 in both cases) whereas no statistically significant differences existed between populations of the other two lineages (*P_adj _*= 0.18). The prediction of the HS for the Central lineage (Figure 9b) showed high values for the Central lineage (>80%) and almost no predictability for the other two lineages (<2%). The predictions for the other two lineages showed highest HS for the modelled lineage, lowest HS for the Central lineage, and intermediate HS for the remaining lineage.

Second, we estimated the overprediction of lumped models compared to the corresponding overlaid split models ([19], see methods) and found that the central clade's niche was the most divergent niche, which is in line with the above findings. Models of the Central lineage lumped with one of the other lineages predicted on average 39.2% more suitable habitat than the overlaid split models (Table 5), which is 8.03% more than overprediction when modelling the *edwardsianus *and Western lineage. Similarly, the difference in false-positive rates between lumped and overlaid split models was on average higher when the Central lineage was included (32.03%) than when comparing the Western and the *edwardsianus *lineage (Table 5). Lumped models including the Central lineage had on average a false positive rate that was 11.31% higher than the one when comparing the *edwardsianus *and Western lineage.

**Table 5:** Estimation of niche divergence using different comparisons of lumped and split models

Compared lineages	% overprediction	false positive rates
*edwardsianus *- Central	37.50	31.62
Western - Central	40.97	32.45
*edwardsianus *- Western	31.20	20.72

% overprediction of lumped models with respect to the prediction of split models is given for each lineage pair. False positive rates were based on presence data of the official Spanish Atlas of Amphibians and Reptiles (Additional File 3: Figure S3).

Finally, our results of ensemble predictions showed that ENFA results of predictive maps, niche divergences and geographic overlaps are robust to inter-model variability arising from different algorithms used for model building (Additional File 2).

## Discussion

Here, we address how geology, climate, and ecology shaped current diversity in *Psammodromus hispanicus *using a multidisciplinary approach including phylogenetic, phenotypic, phylogeographic, and ecological niche analyses.

### Species Status and Phylogenetic Relationships within Iberian *Psammodromus*

We reconstructed congruent mt- and nuclear-based phylogenies (and the corresponding networks) that confidently recovered three major lineages in *P*. *hispanicus*, corresponding to *P*. *hispanicus edwardsianus*, *P*. *hispanicus hispanicus *Central lineage, and *P*. *hispanicus hispanicus *Western lineage. The molecular clock indicates that the age of divergence of the Central and the *edwardsianus *lineages was about 4.8 (1.5-8.7) Mya, which, together with phylogeographic and phenotypic evidence, strongly suggest that these two lineages reflect independent evolutionary units. The divergence between these two lineages and the Western lineage was estimated to have occurred about 8.3 (2.9-14.7) Mya. Altogether, the rather old age of divergence, the lack of haplotype and geographic overlap, as well as the existence of phenotypic differentiation, and ecological niche divergence (in the Central lineage), allow postulating that both the Central and the Western lineage may be valid species.

There is significant phenotypic differentiation among the three lineages. The Western lineage was phenotypically intermediate between the *edwardsianus *and Central lineages, which showed higher phenotypic differentiation. The latter two lineages differed in 11 of the 12 studied traits (Table 3: femoral pores, number of ocelli, SVL, SVL ratio, snout shape, body mass, number of ventral scales, head ratio, number of collar and throat scales, and nuptial coloration) whereas the Western lineage differed from the *edwardsianus *lineage in five traits (femoral pores, number of throat scales, snout shape, head ratio, and nuptial coloration) and from the Central lineage in four traits (femoral pores, number of ocelli and throat scales, and snout shape). The Western lineage showed trait values that were intermediate between those measured in the Central and the *edwardsianus *lineages in 8 of the 12 measured traits (Table 3a). Moreover, all specimens of the *edwardsianus *lineage could be distinguished from the other two lineages by the presence of a supralabial scale below the subocular scale. This finding is in line with previous taxonomy, where its presence has been used to distinguish between the two subspecies, *P*. *hispanicus hispanicus *and *P*. *hispanicus edwardsianus *[20].

For *P*. *algirus*, phylogenetic analyses based on molecular data provide evidence for the existence of four statistically supported lineages, two corresponding to the SW and NW clades of *P*. *algirus *[16], another corresponding to the nominate lineage of *P*. *algirus *(from Africa), and the oldest one corresponding to the Eastern lineage of *P*. *algirus *[16]. The molecular clock showed that the NW and SW clades split only around 1.00 (0.3 - 1.9) Mya, and that the Eastern lineage split around 3.01 (1.0 - 5.5) Mya. The former divergence estimate is similar to the oldest divergence times inferred for the clades within the *edwardsianus *and Central lineage (0.86 (0.3 -1.6) and 0.76 (0.2 - 1.5) Mya, respectively; Figure 3), whereas the latter estimate coincided with the split of the clades within the Western lineage (2.80 (0.9 - 5.1) Mya; Figure 3). The younger datings correspond to the Pleistocene, suggesting a role of glaciations at the origin of the different lineages. Interestingly, there is geographic overlap between northern and southern populations of the Central and the *edwardsianus *lineage, as well as between the SW and NW clades of *P*. *algirus*, suggesting that in both cases reproductive isolation among lineages/clades may exist. Within lineages, the recovered trees and network, as well as population genetic analyses suggest northward expansion of *P*. *hispanicus *from southern refugia [21,22], and incipient and ongoing genetic isolation, while the pattern is less clear within *P*. *algirus*.

### Biogeographic Implications

According to our results, the *P*. *algirus *group likely had an Iberian origin, and first split into eastern and western lineages (Figure 1). Within the western lineage, a second split separated the Iberian ancestor of the Northwestern + Southwestern clades from Moroccan *P*. *algirus*, suggesting that *P*. *algirus *colonized Africa from the Iberian Peninsula. These inferred biogeographic patterns confirm previously reported results based on partial 12S rRNA, 16S rRNA and *cytochrome b *sequences [23]. However, our molecular dating suggests that the Eastern lineage split from the other lineages slightly later than previously estimated (3.6 ± 0.05 mya [23]). Unfortunately, we cannot determine whether the origin of the genus *Psammodromus *was Iberian or North African since our phylogeny did not include two key African species, *P*. *blanci *(likely the sister group of *P*. *hispanicus*; [23]) and *P*. *microdactylus*.

Differentiation of the Western lineage from the ancestor of the other two *P*. *hispanicus *lineages occurred in the Miocene when a progressive uplift started to close the East of the Betic Straits, and formed the Guadalquivir basin (Early Messinian; 7.2-5.5 Mya) [24]. During the same geological period, there were hypothesized splits in several other Iberian reptile and amphibian genera such as *Lissotriton *[25], *Alytes*, [26], and *Blanus *[27], producing in some of them [25 and 27] a similar east-west differentiation pattern. The split between the Central and the *edwardsianus *lineages dates back to the Miocene/Pliocene boundary and thus close to the Messinian salinity crisis and the opening of the Gibraltar Strait. During this period an uplift of the Spanish Central System occurred that led to the current configuration of the Iberian Peninsula's main river drainages [28], indicating that major geologic and climatic changes occurred. Accordingly, Pliocene diversification has been reported for many Iberian groups including freshwater fishes [29-31] and amphibians [32-34].

The spatial distribution of mt *cytb*, *nad4*, nuclear suppressor of SWI4 1, and nuclear clone 17 diversity showed current allopatry for all three *P*. *hispanicus *lineages suggesting a vicariant event at their origin (see above). A decrease in *cytb *diversity with increasing latitude was observed, which likely indicates northward range expansion of all three lineages. The large, negative, and significant test statistics of the neutrality tests in the *edwardsianus *and Central lineage further supported this result. However, statistical support for a northward range expansion in the Western lineage was low, most likely due to the small sample sizes obtained from the southern Peninsula (*N *= 7 individuals belonging to three different haplotypes) and the rather homogenous haplotype distribution on the northern Peninsula (mainly haplotype CE7). Similar patterns were observed in the suppressor of SWI4 1 diversity. The observed range expansions may be the result of post-glacial range expansions [35] from glacial refugia located south of the Guadiana and the Júcar River. According to the molecular clock, range expansions may have occurred around 0.8 (0.2 - 1.5) Mya, which coincides with the Pleistocene glacial and interglacial periods [36]. Similar Pleistocene patterns of range expansion during interglacial periods have been reported for reptile species [37,38] and also for insects, molluscs, amphibians, mammals, and plants [39,40].

### Niche Modelling within *P*. *hispanicus*

We performed ecological niche modelling for each of the three identified lineages to obtain predictive maps of habitat suitability, and assess predictor relevance and niche divergence. The habitat suitability maps fitted the realized niche reasonably well, and the model omission error was only 13.7% with respect to the *P*. *hispanicus *presences cited in the official Spanish Atlas of Amphibians and Reptiles [15] (analysis based on 10 × 10 km resolution) and no sampling bias could be detected.

For all comparisons among the three *P*. *hispanicus *lineages, habitat suitability scores in the sampled populations were significantly different among lineages and highest in the lineage populations for which the distribution was modelled. These results indicate that lineages tend to differ in the optimal portions of their niches. According to the spatial prediction models, the mean temperature of driest quarter was one of the most important predictors of the observed distributions of the Central and the Western lineage. In contrast, mean temperature of wettest quarter, minimum temperature, and precipitation of coldest quarter were the most important predictors of the *edwardsianus *lineage's distribution. This pattern was in line with the finding that the *edwardsianus *lineage inhabited areas with lower vegetation cover on the generally warmer and drier eastern coast of the Iberian Peninsula. The Central lineage inhabited central peninsular habitats characterized by intermediate vegetation cover, precipitation and temperature in wettest quarter, and with the lowest minimum temperatures of coldest quarter and precipitation seasonality. The Western lineage lived in habitats with the highest vegetation cover, winter precipitation, precipitation seasonality, and winter minimum temperatures, and with the lowest temperatures during the wettest quarter, corresponding to the more humid and climatically more stable Western parts of the Iberian Peninsula.

We used different approaches to assess niche divergence between the three lineages, and analyses revealed the same overall pattern. Interpredictivity, differential model overprediction among hierarchical taxonomic groups, and the extent of geographic overlap of the model predictions, showed that the ecological niche of the Central lineage was most divergent, whereas ecological niches of the Western and *edwardsianus *lineages were more similar. Based on the reconstructed tree topologies and following the principle of parsimony, we can infer that the ancestor of the Central and *edwardsianus *lineages likely occupied a niche similar to that of its sister group, i.e. the Western lineage. Hence, niche divergence occurred during the evolution of the Central lineage.

There is suitable climatically suitable habitat for the *edwardsianus *lineage in the west of the Guadalquivir River, and suitable habitat was predicted for the Western lineage on the Eastern Iberian Peninsula (Figures 8a and 8c). The spatial predictions showed important overlap between the *edwardsianus *and the Western lineage on the southern and southwestern Iberian Peninsula (Figure 8e), where both lineages share potentially suitable ecological conditions on both sides of the Guadalquivir River. This suggests that a barrier between Málaga and the Guadalquivir River may prevent population mixing and led to vicariant diversification by impeding dispersal, from the betic uplift in the late Miocene/early Pliocene until the present. Earlier findings in amphibians (e.g. *Discoglossus galganoi *and *D*. *jeanneae *[41]) are in agreement with this hypothesis.

When comparing the Central lineage with the *edwardsianus *lineage, suitable habitat for both lineages was located in the centre of the Iberian Peninsula (Figure 8d), and there was no connection through HS values in the estimated contact zone (Figure 8a and 8b). In contrast, the Central lineage showed almost no habitat overlap with the Western lineage (and thus, the Central-*edwardsianus *ancestor) (Figure 8f), which indicates that niche divergence may be an important force preventing the mixing of these lineages.

In summary, these results show that the Western lineage and the ancestor of the Central and *edwardsianus *lineages may have been geographically isolated due to a barrier [41] that still may prevent mixing of the Western and *edwardsianus *lineages, and that niche divergence may have played a limited role in the separation of these two lineages. In contrast, our analyses provide evidence that niche divergence was more prominent in the Central lineage, potentially preventing gene flow with its sister lineage.

### Integrating Phylogenetic, Phenotypic, Geological, and Environmental Data

Determining the relative role of historical and ecological factors as evolutionary drivers of diversification is a central question in evolutionary biology. In this work, we performed a multidisciplinary approach to delimit current diversity of *P*. *hispanicus*, and to understand its origin and maintenance. Phylogenetic (mt, nuclear, and combined) and phenotypic data allowed us to differentiate three lineages, which showed important differences in phenotypic traits. The early splitting of the Western lineage may coincide with a vicariant event, which is the initiation of the betic uplift and the formation of the Guadalquivir basin at the end of the Tortonian about 7 Mya, but the large confidence intervals hinder more precise dating. Interestingly, ecological niche modelling shows large overlap in suitable habitat between the Western and the *edwardsianus *lineage, which contrasts with the phylogeographic *cytb*, *nad4*, suppressor of SWI4 1, and clone 17 haplotype distribution that show no spatial overlap of the two lineages. The molecular clock together with genetic, geological, and niche-modelling data suggest that an event related to the betic uplift at the Miocene-Pliocene boundary hindered until present gene flow of *Psammodromus*. The important niche overlap together with the limited gene flow supports a diversification model of niche conservatism in allopatry for the early divergence of the Western lineage and the common ancestor of the Central and the *edwardsianus *lineage.

The split between the Central and the *edwardsianus *lineage in the Early Pliocene coincides with the uplift of the Spanish Central System, which resulted in a change of the drainage patterns from internal to external, and the forming of the present river systems in the Iberian Peninsula [42]. These geological changes produced major climate changes ranging from dry climate during the Messinian salinity crisis to more humid habitats in the early Pliocene. These climate changes may be responsible for diversification within *P*. *hispanicus*. The finding that the ecological niche of the Central lineage was most divergent with respect to those of the *edwardsianus *and Western lineages (despite the older age of the Western lineage split), implies that niche divergence and ecologically-based divergent selection were involved in the diversification process. However, since the Central lineage diverged from the *edwardsianus *lineage when major climatic changes happened, the possibility that a climatic barrier and initial niche conservatism could have been responsible for the initial splitting cannot be fully discarded [43].

The large overlap of suitable habitat between the *edwardsianus *and the Western lineage compared to the small overlap with the Central lineage should be reflected in those phenotypic traits that are adaptive. We explored two phenotypic traits that may be under natural selection in lizards (coloration and number of throat scales). Vegetation cover may determine which colours are cryptic and which ones are conspicuous, and thus, background matching to avoid predation may be the cause for the evolution of colour differences [44,45]. The Western and the Central lineages do not differ in their nuptial coloration, which is greener than that of the *edwardsianus *lineage. A second example is the number of throat scales. Since smaller scales and more numerous scales reduce skin water exchange [46], differences in the number of throat scales may have evolved due to precipitation differences. Here we found that the *edwardsianus *lineage shows an increased number of throat scales with respect to the Western lineage, whereas the Central lineage shows the lowest number of throat scales. When comparing the ecological scenarios derived from phenotypic traits (Table 3) with real differences in environmental parameters (Table 4), there was a general lack of correlation between environmental parameters and known phenotypic traits under selective pressure, which is in line with previous findings that phenotype does not necessarily predict ecology. Thus, our results suggest that the use of phenotypic traits as a surrogate for ecology in studies dealing with phylogenetic niche conservatism may be problematic [11], but see [47]. The complexity in phenotypic variation found here, encourages future studies that aim at partitioning phenotypic variation into independent contributions of ecology, phylogenetic inertia, and phylogenetically structured ecological variation, as proposed for higher taxon levels [48].

## Conclusions

Our results indicate that divergence due to both historical geographic barriers and environmental differences may have led through time to the evolution of three *P*. *hispanicus *lineages, and that these processes are still acting now to prevent population mixing over the largest part of their allopatric distributions. Our preliminary results on the phylogeographic patterns observed in *P*. *algirus *suggest that similar patterns may also exist in other related lizard species, as previously suggested. Here, we highlight the importance of taking a multidisciplinary approach for disentangling the relative roles of vicariant and adaptive divergence in generating currently observed biological diversity. We found that a vicariant event was at the origin of the first splitting event, which was followed by a second splitting event (split between *P*. *hispanicus hispanicus *Central and *P*. *hispanicus edwardsianus *lineage) in which the role of ecologically based divergent selection (i.e. niche divergence) may have been more prominent. This indicates that diversity in a lineage is the result of different temporally separated evolutionary processes, which is concordant with patterns observed in other groups (e.g. cichlids, [49]; Gobiidae, [50]; Passerine birds, [51]; anoles, [52,53]).

## Methods

### Samples and DNA Extraction

We conducted a systematic population sampling all over Spain (one population per intersection of a ± 250 km grid) and captured a total of 285 specimens between April and May 2006. The capture and handling of lizards was conducted under the licenses provided by Junta de Andalucía, Gobierno de Aragón, Junta de Castilla y León, Junta de Comunidades de Castilla - La Mancha, Generalitat de Catalunya, Junta de Extremadura, Xunta de Galicia, Comunidad de Madrid, Gobierno de Navarra, Generalitat Valenciana, Parque Natural de l'Albufera (Valencia), Parque Natural del Delta del Ebro (Cataluña), Parque Nacional de Doñana (Huelva), and Gobierno de España. Of the 285 captured specimens, 265 were identified as members of *P*. *hispanicus*, whereas 20 specimens were identified as members of *P*. *algirus*. We were able to collect individuals in 11 previously known populations (50%; see Additional File 1), failed to find any sample in three known populations, but found samples in adjacent unknown populations (13.6%; [15]). We also screened potential habitats in locations where no records existed previously, and were able to successfully collect specimens in eight yet unknown populations (36.4%). Sample locations are shown in Figure 4, and the location, sample size and collection numbers are given in Additional File 4.

For each captured individual, we collected a small piece of the tail tip, which was preserved in 70% ethanol at -20ºC. Genomic DNA was extracted from ethanol-preserved tissues using the ChargeSwitch gDNA Micro Tissue Kit (Invitrogen). All individuals were sequenced for mt *cytochrome b *(*cytb*) gene, and a subsample including 56 representative specimens of *P*. *hispanicus *and 16 specimens of *P*. *algirus *was also sequenced for mt *NADH dehydrogenase subunit 4 *(*nad4*) gene, and two nuclear loci (see Additional File 4 for specimen number and GenBank accession numbers).

Two primer combinations (L14841 [54] + H15149 [54], and MNCN-Glu F [55] + H15149 [54]) were used to amplify part of the *cytb *gene in different individuals, yielding sequence lengths between 275 and 418 bp (depending on the specimen). A fragment of 865 bp of the *nad4 *gene was amplified using primers *nad4 *and LEU [56] for most specimens of the subsample. For specimens of the subsample where we could not amplify the *nad4 *fragment, we designed two additional primers (forward: L11162, reverse: tRNA-His H11749, see Table 6) in conserved regions of the *nad4 *gene to amplify a shorter and fully overlapping region (504 bp). Two nuclear regions were obtained by cloning. Genomic DNA of *P*. *hispanicus *was digested with *Bam*HI and *Bg*lII restriction enzymes, cloned into pBlueScript SK+ vector, and transformed into *E*. *coli *XL10 strain (Stratagene) following standard protocols [57]. Plasmids were purified from positive white clones, and sequenced with universal M13 primers in an automated DNA sequencer (ABI PRISM 3700, Applied Biosystems) using the BigDye Terminator v3.1 Cycle Sequencing Kit, and following manufacturer's instructions. Sequence similarity searches [58] were performed against GenBank databases using both the *nucleotide blast *and the *blastx *algorithms. The best hit for one of the loci was *Anolis carolinensis *suppressor of SWI4 1 homolog (percent coverage = 99%, e-value ≤ 3.0E−110, maximum identity ≥ 71%), whereas no relevant matches were obtained for clone 17. For partial suppressor of SWI4 1 gene, the forward 8F17-F35 and the reverse 8F17-R815 primers were designed to amplify a fragment of 886 bp (see Table 6). For clone 17, a total of three forward primers (17-F20, 17-F32 and 17-F53), and two reverse primers (17-R613 and 17-R749) were designed to amplify fragments of 372 - 977 bp in length (see Table 6).

**Table 6:** List of newly developed primers used for PCR and DNA Sequencing

Gene	Primer name	Sequence
*nad4*	L11162	5'-CGACAAACAGAYCTAAAAGC-3'
*nad4*	tRNA-His H11749	5'-TCTAGAGTCACAATCTAGTGT-3'
clone 17	17-F20	5'-CAGTTACTTAGATCAATGGACGGTT-3'
clone 17	17-F32	5'-TCAATGGACGGTTTCAGCAA-3'
clone 17	17-F53	5'- GCTGTACAGTTCTAGGTTTTGCT-3'
clone 17	17-R613	5'- TCAAGGCAGAGATACTAATGGAG-3'
clone 17	17-R749	5'- TGTGGGCTTTACATCAGAAGTACC-3'
suppressor of SWI4 1	8F17-F35	5'-GGGAACGGCCTTGCCATCTA-3'
suppressor of SWI4 1	8F17-R815	5'-TGGAATCCTCTGCAGCAATATTC-3'

PCR amplifications were conducted in 25 µl reactions containing 67 mM Tris- HCl, pH 8.3, 1.5 mM MgCl_2_, 0.4 mM of each dNTP, 2.5 µM of each primer, template mtDNA (10-100 ng), and *Taq *DNA polymerase (1.5 U, Roche). For the PCR amplification of the *cytb *gene fragment, an initial 60 s denaturing step at 93º C was followed by 35 cycles of denaturing at 93º C for 60 s, annealing at 45-50º C for 60 s, and an extension phase at 72º C for 60 s. The final extension phase at 72º C lasted for 6 min. PCR cycling conditions for amplifying the *nad4 *fragment were: cycle 1 (94º C for 60 s, 72° C for 60 s), cycle 2 - 36 (94º C for 60 s, 50-58º C for 60 s, 72º C for 60 s), cycle 37 (72º C for 6 min). PCR cycling conditions for amplifying partial suppressor of SWI4 1 gene were: cycle 1 (94º C for 60 s, 72° C for 60 s), cycle 2 - 41 (94º C for 60 s, 53.5-59.5ºC for 60 s, 72º C for 60 s), and cycle 42 (72° C for 6 min). Those for amplifying locus 17 were: cycle 1 (94º C for 60 s, 72° C for 60 s), cycle 2 - 41 (94º C for 60 s, 53-66º C for 60 s, 72º C for 60 s), and cycle 42 (72° C for 6 min).

PCR products were checked in 1.5% agarose gels, purified by standard ethanol precipitation, and sequenced in an automated DNA sequencer (ABI PRISM 3700, Applied Biosystems) with the corresponding PCR primers using the BigDye Terminator v3.1 Cycle Sequencing Kit, and following manufacturer's instructions.

### Phylogenetic Analyses

Three sequence data sets were analyzed: the first data set (mt data set) included *cytb *gene partial sequences of *P*. *hispanicus *(285 specimens), and of seven individuals of *P*. *algirus *(accession numbers: DQ150367, DQ150366, DQ150365, DQ150364, DQ150363, DQ150362, AF206535) obtained from GenBank. In addition, *cytb *sequences of three individuals of *Gallotia caesaris caesaris *(AY151843, AY154903, AF439948) and three individuals of *Gallotia caesaris gomerae *(AY151842, AY154902, AY154901) were used as outgroup taxa, and for molecular clock calibration. The second data set (nuclear data set) included partial sequences of two nuclear loci (suppressor of SWI4 1 and clone 17) of a subset of 56 individuals representing the major lineages of *P*. *hispanicus *(as per previous mt analyses), 16 individuals of *P*. *algirus*, and six specimens of *G*. *caesaris*. A total of 16 individuals were heterozygotes in 3.8% of their nucleotide positions, which were coded as N in all subsequent analyses. The third data set (combined data set) included *cytb*, *nad4*, and the two nuclear loci (suppressor of SWI4 1 and clone 17) for the subset of 54 individuals representing the major lineages of *P*. *hispanicus*.

Sequences were aligned using Clustal × version 1.83 [59] with default penalties for gap opening and gap extension, and alignments were visually verified. For each molecular marker, independent alignments were prepared, and the best-fit models of nucleotide substitution were inferred using the Akaike information criterion (AIC; [60] as implemented in Modeltest version 3.7 [61]). The mt, nuclear, and combined data sets were analyzed using maximum likelihood (ML; [62], and Bayesian inference (BI; [63]. ML analyses were performed with RAxML version 7.2.6 [64] using the rapid hill-climbing algorithm [65] and starting from 100 distinct randomized maximum-parsimony starting trees. For BI analyses, we used MrBayes version 3.1.2 [63,66]. We ran four simultaneous Markov chains for 20 million generations, sampling every 2000 generations (10,000 trees), and discarding the first 10% of generations (1,000 trees) as burn-in to prevent sampling before reaching stationarity. Adequate convergence of the Bayesian Markov chain Monte Carlo runs was assessed using Tracer version 1.5 (http://tree.bio.ed.ac.uk/software/tracer/). Two independent BI runs were performed to increase the chance of adequate mixing of the Markov chains, and to give some chance of spotting failure to converge. For both ML and BI analyses, three partitions were used for the mt data set (accounting for each codon position), two partitions were used for the nuclear data set (one per locus), and eight were used for the combined data set (accounting for each nuclear marker, plus each codon position of each mt marker). For BI analyses, independent best-fit models of nucleotide substitution (as selected by Modeltest) were used for each partition with model parameters unlinked and estimated separately among partitions. For ML analyses, the GTR + Γ model was used for all partitions due to software (RAxML) constraints, and model parameters were unlinked and estimated separately among partitions. Statistical support for internal branches in the ML analyses was evaluated by non-parametric bootstrapping [67] with 2,000 replicates and using posterior probabilities in the BI analyses.

### Dating of Divergence Times

The combined dataset was used to date major cladogenetic events within the *Psammodromus *phylogeny using the Bayesian relaxed clock method [68] as implemented in BEAST version 1.6.1 [69]. This widely used method for dating phylogenies [70] assumes a relaxed uncorrelated clock with rates drawn from a lognormal distribution across branches. The ML optimal topology was used as a starting tree, and the birth-death process [71] was used to describe diversification. The partitions used for BI of the combined data set and corresponding models (see above) were employed for the dating analysis. A first run of 10 million generations was first performed to optimize the scale factors of the prior function. The final Markov chain was run twice for 100 million generations, sampling every 10,000 generations, and burn-in and convergence of the chains were determined with Tracer. Effective Sample Size (ESS) values were over 350 for all parameters sampled.

Time estimates were calibrated using the formation of El Hierro 1.12 ± 0.02 Mya [72] as internal time constraint (maximum age) for the split between *Gallotia *lizards from El Hierro and La Gomera (Canary Islands). The time constraint was used as 'soft' bound [73]: the mean and standard deviation of the normal distribution were chosen so that the mean is the arithmetical mean of the interval, and 95% of the probability lies within the lower and upper bound. Analyses using a uniform instead of a normal distribution yielded almost the same average time estimates and confidence interval width (not shown).

### Genetic Structure

Population genetic analyses of *P*. *hispanicus *were performed using Arlequin 3.11. [74]. Minimum-spanning networks based on *cytb, nad4*, suppressor of SWI4 1 and clone 17 haplotypes were inferred independently. To infer population dynamics from *cytb *haplotype data, each of the three major lineages of *P*. *hispanicus *(as recovered in the phylogenetic trees and the minimum-spanning networks) was split into northern and southern populations (approximately above and below 40ºN separating the three lineages into approximately 50% northern and 50% southern populations; see Additional File 1). Descriptive statistics including haplotype diversity (Hd; [75]) and nucleotide diversity (π; [75]) were determined for northern and southern populations, respectively. Inter-haplotype levels of divergence between northern and southern populations were estimated using the fixation index Φ_ST _[76], which includes information on mitochondrial haplotype frequency [77], and genetic distances (TrN93; [78]) with gamma correction. Significance of pairwise population comparisons was tested by 20,000 permutations. An analysis of molecular variance (AMOVA) was used to examine the amount of genetic variability partitioned within and among populations [76]. AMOVA tests were organized in a hierarchical manner so that population structure was studied at increments of increasing spatial scale, which range from structure within and among different populations to northern versus southern groups. Permutation procedures (N = 20,000) were used to construct null distributions, and test the significance of variance components for each hierarchical comparison [79]. In all instances with multiple tests, p-values were adjusted using the sequential Bonferroni correction [80].

### Phenotypic Data

Immediately after capture, standardized digital photographs of each lizard belonging to *P*. *hispanicus *were taken according to the methods used by Fitze & Richner [81]. In brief, living lizards were placed in a box covered with a photographic filter lens to immobilize the individual. This box was placed in an opaque camera box where two flashes were mounted. The settings of the camera and flashes were always identical and the distance between the objective and the object was fixed. Thus all photographs received a standard light exposure. Standard white chips (Kodak Colour Control Patches with R = 255, G = 255, B = 255) were fixed to each side of the filter for detecting potential errors in light exposure and allowing for calibrating the sizes. Photos were taken of the lizards' belly, back and flanks. Lizards were weighed to the nearest 0.001 g, snout-to-vent length (SVL) and total length were measured to the nearest 1 mm, and the number of femoral pores was counted.

Photos were imported into IMAGEJ program (National Institute of Science, USA) and a set of 11 different phenotypic and biometric traits were measured: head length, head width, snout width, snout length, anal scale width, number of ventral scales, presence of a supralabial below subocular, number of throat and collar scales, number of ocelli, and the nuptial coloration (for detailed description, see Table 7). Head shape was calculated by dividing head width by head length [82], and snout shape was estimated by dividing snout length by snout width. Smaller head shape values thus indicate that the head was more pointed, and smaller snout shape values indicate that the snout was less pointed. SVL ratio was calculated by dividing total length by SVL [83], and the relative anal scale width by dividing anal scale width by SVL [84]. The measures of the different phenotypic traits were highly repeatable ([85] statistics based on two blindly taken repeated measurements: the number of throat scales had the lowest repeatability: *F*_10,11 _*= *4.13, *P = *0.014, *r = *0.61; rest of the traits: *F*_10,11 _> 6.52, *P *< 0.002, *r >*0.73, mean *r = *0.86 ± 0.03).

**Table 7:** List and brief description of the phenotypic measurements taken

Variable (unit)	Description
SVL (mm)	snout to vent length
Total length (mm)	snout to tail tip length
SVL ratio	SVL/total length
Body mass (g)	
Head length (mm)	distances between the tip of the snout and the occipital edge
Head width (mm)	distances between the borders of the outermost left and right
	supraocular scales (located behind the eyes).
Head shape	degree of head sharpness. Head width/head length [82]
Snout width (mm)	distance between the left and right foremost intersection point of
	the first supraocular and the first supraciliar scale
Snout length (mm)	distance between the tip of the snout and the orthogonal
	intersection with the snout width
Snout shape	degree of snout sharpness. Snout length/snout width
Anal scale width (mm)	distance between the posterior borders of the anal scale
Relative anal scale	anal scale width/SVL [84]
width	
Femoral pores	mean number of right and left femoral pores
Ventral scales	number of longitudinal ventral scale rows
Subocular scales	number supralabial scales below subocular scale [103]
Throat scales	number throat scales
Collar scales	number well-differentiated collar scales
Number of ocelli	mean number of left and right ocels
Nuptial coloration	sum of presence/absence (1/0) of green coloration on neck, belly, subocular and supralabial scales + number of green coloured longitudinal lines + number of green coloured longitudinal lines that spread further than the middle of the body/2

### Statistics used for the Analyses of Phenotypic Data

All statistical analyses were conducted using R 2.7.0 software (Free Software Foundation, GNU Project, Boston, MA, USA). A total of 211 adults were used for the multivariate analyses. We applied a permutational MANOVA (NP - MANOVA) based on distance measures [86] to investigate differences between lineages revealed by phylogenetic analyses. A total of 9,999 permutations were conducted, following Manly [87]. We first standardized the data in order to avoid differential impact of unequally scaled variables on the posterior analysis [88]. Thereafter, we calculated the dissimilarities between observations based on Euclidean distances, and applied a NP - MANOVA (Adonis function in Vegan package). Results from paired contrasts between lineages were corrected using Bonferroni procedures, indicated as *P_adj _*[80]. The assumption of homogeneity of multivariate dispersion between lineages was fulfilled for all presented analyses [86].

Discriminant functions were derived from the linear combinations of the variables [88,89], to assess the relative contribution of each variable to the differences between lineages and to visualize multivariate differences between lineages.

We also run univariate ANOVAs on each response variable separately in order to understand which traits differed among lineages. All assumptions were verified and, if necessary, transformations or non-parametric analyses were applied. Model assumptions were fulfilled in all cases.

### Ecological Niche Modelling

To obtain predictive maps for the potential distributions of each *P*. *hispanicus *lineage and assess the degree of ecological divergence among lineages, we performed species distribution modelling based on the ecological niche concept [90]. We built models based on environmental predictors and the lineage presences resulting from the phylogenetic analyses (9, 5, and 8 sampled populations for the *edwardsianus*, Central, and Western lineage respectively; Additional File 1).

#### Environmental predictors

We used biologically relevant environmental variables at a 1-km resolution. We initially considered 18 climatic, one topographic and two vegetation index variables, all of them typically being used in biogeographic models as direct and/or indirect predictors of species distributions (e.g. [91-93]). Climatic and topographic variables were obtained from the Worldclim source [94], whose raster maps were downloaded at the EDIT Geoplatform http://edit.csic.es/. As a measurement of primary productivity we used monthly maps of Enhanced Vegetation Indexes (EVI) generated from satellite MODIS images available at the NASA-LP DAAC web page (https://lpdaac.usgs.gov/lpdaac). EVI is an improvement of Normalized Difference Vegetation Index that minimizes canopy background variations and maintains sensitivity over dense vegetation conditions. Besides predicting primary productivity, EVI is also a measurement of shade availability, which in turn, may modulate direct climate effects in ectotherms (e.g. [95]). We generated year-averaged monthly values of EVI for each cell from the oldest period available to the year of taxon sampling (2000-2006). Thereafter, we calculated for each cell the minimum and maximum values over all months.

To meet model assumptions, environmental variables were Box-Cox transformed. Redundancy and colinearity between variables was analysed using Pearson correlations. Eight variables were excluded from the analyses because they were highly correlated with other variables (*r *> 0.90). A total of 13 variables were used for the subsequent analyses: seven temperature predictors (annual mean temperature, mean temperature of wettest quarter, mean temperature of driest quarter, minimum temperature of coldest month, maximum temperature of warmest month, annual temperature range and isothermality), four precipitation predictors (annual precipitation, precipitation of the warmest quarter, precipitation of the coldest quarter and precipitation seasonality), one topographic predictor (elevation) and one EVI index (minimum EVI index).

#### Model building

We built GIS-based models to estimate each lineage's multidimensional niche. We geo-referenced the sampled populations and used digital maps of environmental variables. To obtain habitat suitability (HS) maps for each lineage we used the Ecological Niche Factor Analysis (ENFA) implemented in the GIS-statistical tool Biomapper [96]. ENFA allows the calculation of HS scores for each cell in a gridded map and it is especially suited if absence data are not available, unreliable, or meaningless. ENFA is analogous to principal component analysis with the difference that it is based on the niche concept of marginality (defined as the ecological distance between the lineages optimum and the average habitat within the study area) and specialization (the ratio of the ecological variance in average habitat to that associated to the focal lineage [96]). Environmental variables are compacted into a few factors where the first factor maximizes the marginality and the other factors maximize the specialization of the focal lineage. Finally, those factors that explained the biggest part of variance (i.e. those that best explained a lineage's ecological range) were used to obtain HS scores for each cell in the map ranging from 0 to 100. The distribution of the eigenvalues was compared with the MacArthur's broken stick distribution to decide which subset of factors was used for HS map computation [96]. In addition to the predictive maps based on continuous HS scores, we obtained binary maps reclassified as predictions of suitability and non-suitability. The threshold applied to transform continuous into binary maps was the minimum training presence (the lowest HS scores associated to the populations of each lineage), which classified as suitable areas those corresponding to scores bigger than zero. This method is conservative since it may include cells where the HS scores of lineages are small.

#### Evaluation of models

We evaluated the predictive capacity of our niche models using an independent data set, i.e the *P*. *hispanicus *species distribution published in the official Spanish Atlas of Amphibians and Reptiles [15]. The binary predictions derived for the three lineages were superimposed to obtain an overall prediction for *P*. *hispanicus*. We thereafter calculated the omission error with respect to the known *P*. *hispanicus *presences (% cells with predicted absences, with respect to the number of cells with Atlas presences).

A crucial assumption for assessing niche divergences and overlaps among lineages is that the predictive capacity should be similar for the compared lineages. We therefore investigated the robustness of the lineage specific model predictions, using a jack-knife methodology (e.g. [97]). In brief, we run an individual lineage model with one sampled population excluded (*N*_lineages _- 1) and calculated the proportional prediction difference ((HS*_N _*_lineages _- HS*_N _*_lineages-1_)/HS*_N _*_lineage_). This procedure was repeated for each population. To evaluate potential biases between lineages we used a one-way ANOVA with the proportional prediction difference as the dependent variable and lineage as a factor.

#### Assessment of environmental predictor relevance

First, we analyzed differences in environmental predictors between lineages of *P*. *hispanicus *using one-way ANOVAs. These analyses allow linking phenotypic differences between lineages with differences in potential selective pressures. Second, we estimated for each lineage the relevance of each environmental predictor using ENFA. For each environmental predictor, we determined the highest predictor value among ENFA factors that explained an important part of the variation. Factor importance was determined using the broken stick distribution. Environmental predictor relevance increases with increasing predictor values (Figure 7; for further details see [98]).

#### Assessment of ecological niche divergence within P. hispanicus

We applied different principles to assess divergence of niches between lineage pairs, and examined whether there is concordance among procedures. First, we estimated interpredictivity among lineages [99,100]. We used ecological niche models derived for a given lineage and predict all sampled populations (Figure 9). High predictability for the populations belonging to the lineage for which the niche model was constructed and low predictability for populations of the other lineages (i.e. low interpredictivity) indicates divergent ecological niches. We calculated interpredictivity among lineages using continuous HS scores and binary predictions (see Model building) and used one-way ANOVAs based on angularly transformed scores [88] for HS scores to compare predictability between lineages. *Post-hoc *comparisons were performed using Tukey´s range test.

Second, we compared niche models based on split and lumped taxonomic groupings following Raxworthy et al.'s rational [19]. We calculated the excess of prediction (area predicted by lumped model - area predicted by superposed split models) for each lineage pair and investigated whether the combined ecological niche model predicts more suitable area than the superposed split models. We also calculated the false-positive rates of lumped and superposed split models by determining the percentage of predicted niche space, where now presences have been cited [15] (wrongly predicted presences/total of absences). Since the Atlas does not discriminate among lineages, we estimated the false-positive rates excluding Atlas presence data within the minimum-convex polygon of the not considered lineage. To estimate niche divergence we calculated differences in false-positive rates for each lineage pair (false positive rate of lumped - split models). Bigger false-positive rate differences indicate higher niche divergence.

Finally, the extent of geographic overlap of habitat suitability maps between lineage pairs can reveal information about niche divergence and about the availability and spatial structure of the shared environmental conditions. To estimate overlap, we used binary maps (see model building) and determined areas suitable for two lineages (Figures 8c, d, and 8e). To visualize the spatial structure and the connectivity of shared environmental conditions, we first localized potential lineage contact zones using lineage-specific minimum-convex polygons (Additional File 3 - Figure S3) and considered areas as potential contact zones where two polygons overlapped. Zoomed contact zones of lineage specific predictions were provided (Figures 8a, b, and 8c), that show the extent to which lineages are isolated/connected through continuous HS scores.

#### Assessing inter-model variability with respect to ENFA

To understand whether the main conclusions derived using ENFA were robust, we assessed inter-model variability using MaxEnt, Multidimensional Envelope (MDE). We examined to which extent niche divergence and geographic overlaps may depend on the used algorithms, and obtained ensemble predictions [101], which join the results of the three modelling techniques (ENFA, Multidimensional Envelopes and MaxEnt). For more detailed information about the applied techniques and analyses, see Additional File 2.

## Authors' contributions

PSF and RZ conceived the study and obtained funding. DSM, LMSJ, PA, VGJ, and PSF collected the specimens. LMSJ and PSF collected and analysed the phenotypic data. VGJ and PSF carried out DNA sequencing. TS and VGJ developed the nuclear markers. DSM and PSF conducted genetic analyses. PA performed ecological niche models. All authors discussed the implications of the results, and PSF drafted the manuscript. All authors commented the manuscript and approved its final version.

## Funding

LMSJ was supported by a PhD fellowship (I3P 060501) from the Consejo Superior de Investigaciones Científicas (CSIC) of Spain co-financed by the European Social Fund, VGJ was supported by a PhD fellowship from the Ministry of Science and Innovation of Spain (FPU AP2006-01678), DSM was supported by a postdoctoral fellowship of the Ministry of Science and Innovation of Spain (MEC/Fulbright 2007-0448), and PSF by a grant from the Ministry of Science and Innovation of Spain (Programa Ramón y Cajal, RYC-2003-006136). The project costs were financed by a grant from the Comunidad de Madrid, Spain (200530M090 to PSF and RZ).

## Supplementary Material

Additional file 1 **Table S1. Sampling locations and obtained sample size**. A. Samples of *P*. *hispanicus*, b. samples of *P*. *algirus*. Given are the population number, which corresponds to the populations shown in Figure 4, the locality, Spanish province, the exact location, the sample size (number males/number females/number individuals with unknown sex), the total sample size per population and the collection numbers. We further indicate whether the authors knew the population previously (yes: known population; close: a population close by was known, no individuals were found, and thus, the search radius was extended and individuals in an unknown location were found; no: the population was not known), whether the record is new with respect to the Atlas of Amphibians and Reptiles [15], and whether the population was located on the northern (N) or southern (S) Peninsula. Click here for file

Additional file 2 **Assessment of inter-model variability with respect to ENFA**. Supporting methods, results and figures are provided. The supporting information includes predictive maps of *P*. *hispanicus *lineages and spatial projections of niche overlap derived from lineage specific ensemble forecasts (Figure S1) using combined models of ENFA, MaxEnt and Multidimensional Envelopes (MDE), percentage of niche overlap predicted by different types of ensembles and by ENFA (Table S2), and differences between lineages in habitat suitability of sampled populations predicted by MaxEnt (Figure S2). Click here for file

Additional file 3 **Figure S3. Minimum-convex polygons of *P*. *hispanicus *populations**. Minimum-convex polygons encompassing the populations of the Western (green), Central (red), and *edwardsianus *lineage (blue). Overlap between polygons denotes potential contact zones between lineages. Click here for file

Additional file 4 **Table S3. Sampled specimen**. Collection number, GenBank accession numbers, and MNCN/ADN voucher numbers of the specimens used in this study. Click here for file
