# Supplementary material for: Integrative analyses of speciation and divergence in Psammodromus hispanicus (Squamata: Lacertidae)
Source: BMC Evol Biol. 2011 Nov 30;11:347. doi: 10.1186/1471-2148-11-347 (PMC3293786; doi:10.1186/1471-2148-11-347)
Supplement: Additional file 2 — Assessment of inter-model variability with respect to ENFA. Supporting methods, results and figures are provided. The supporting information includes predictive maps of P. hispanicus lineages and spatial projections of niche overlap derived from lineage specific ensemble forecasts (Figure S1) using combined models of ENFA, MaxEnt and Multidimensional Envelopes (MDE), percentage of niche overlap predicted by different types of ensembles and by ENFA (Table S2), and differences between lineages in habitat suitability of sampled populations predicted by MaxEnt (Figure S2). [file 1471-2148-11-347-S2.PDF]

Additional File 2. Assessing inter-model variability with respect to ENFA

1. Predictive maps and geographic overlap of habitat suitability using combined models of ENFA, MaxEnt and Multidimensional Envelopes (MDE)

To assess to which extent ENFA models (see main text) are robust regarding inter-model variability, we compared predictive maps obtained by ENFA with maps obtained from combined models of three different modelling techniques: ENFA, MaxEnt and Multidimensional Envelope (MDE).

MaxEnt is a widely used method based on maximum entropy to generate outputs of area favourability for a given attribute [1]. MDE is the simplest method for characterizing niches and estimates the environmental range (the hypervolume encompassed by the minima and maxima) of the recorded presences [2]. The use of MDE is recommended when characterizing the fundamental rather than the realized niche [3]. It is important to realize that all methodologies suffer from shortcomings when characterizing the fundamental niche using occurrence data [3-5]. Since MDE gives equal weights to all predictors we used a previously developed protocol to obtain the most relevant predictors with ENFA and include them in the MDE models [6]. Thus, we included in MDE models those predictors that fell within the three upper quartiles of the most relevant predictors in ENFA models, which also fell within the three upper quartiles of predictor importance in MaxEnt.

All continuous predictive maps derived from ENFA and MaxEnt were transformed to binary maps using as a threshold the minimum training presence. Ensemble forecasting has been suggested to reduce inter-model uncertainty [7]. Therefore, we overlaid predictive maps derived from ENFA, MDE, and MaxEnt and generated predictive maps, where only cells predicted to be favourable by at least two models were considered. Thereafter, we generated pairwise spatial projections of niche overlap between lineage pairs.

Figures S1a, S1b, and S1c showed that lineage specific ensemble forecasts were similar to those produced by ENFA (Figures 8a, 8b, and 8c of the main text). Moreover, the spatial projection of niche overlap between lineage pairs (Figures S1d, S1e, and S1f) showed that the spatial patterns were proportional to those obtained by ENFA (Figures 8d, 8e, and 8f of the main text).

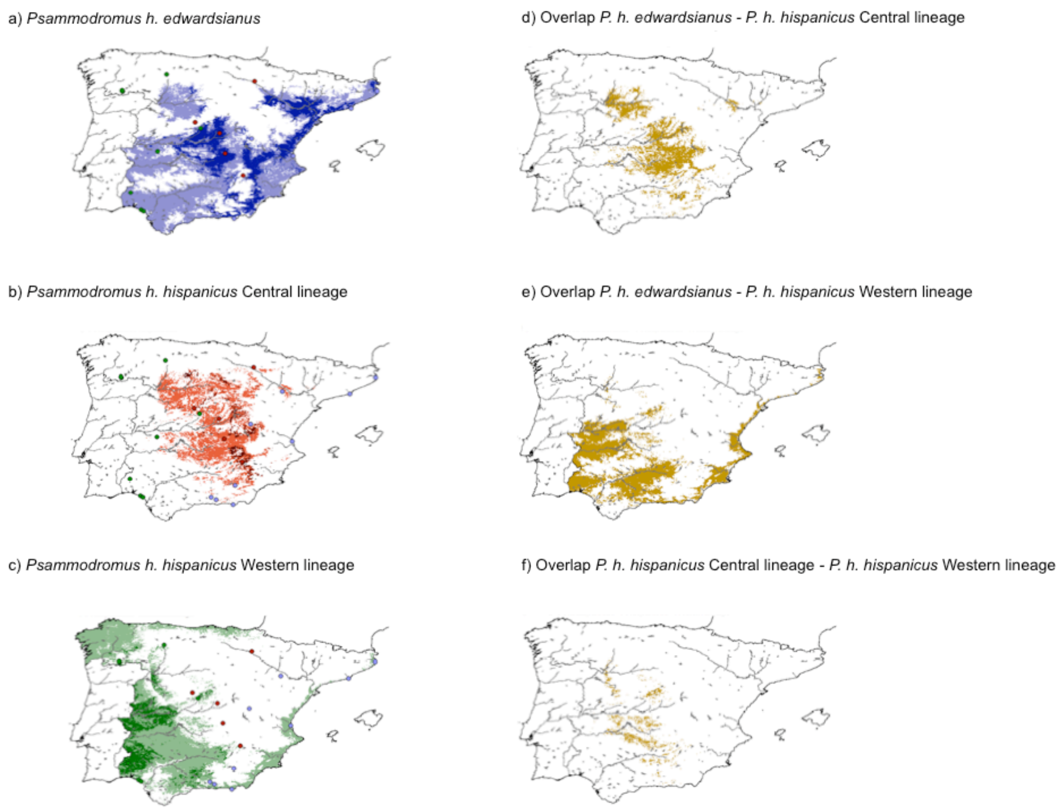

Figure S1. Predictive maps of *P. hispanicus* lineages derived from lineage specific ensemble forecasts. Shown are habitat suitability maps for (a) *edwardsianus* lineage, (b) Central lineage, and (c) Western lineage. Color intensity denotes coincidence of two or three model predictions. Sampled localities are indicated in blue (*edwardsianus* lineage), red (Central lineage), and green (Western lineage). Spatial projections of niche overlap between lineage pairs are given in the right panels (d, e, f).

To examine whether spatial overlap estimated by ENFA was proportional to different types of ensembles we calculated the relative extent of spatial overlap based on predictions generated by:

1) ensembles based on MDE that included the three upper quartiles of predictors (ten predictors), whose relevance was estimated using ENFA, and including suitable habitat predicted in at least two models (MDE, Maxent, or ENFA).
2) ensembles based on MDE that included the first half of predictor (seven predictors), whose relevance was estimated using ENFA, and including suitable habitat predicted in at least two models.
3) ensembles based on MDE that included the three upper quartiles of predictors (ten predictors), whose relevance was estimated using ENFA, and including suitable habitat predicted in all three models (MDE, Maxent, or ENFA).
4) ensembles based on MDE that included the first half of predictor (seven predictors), whose relevance was estimated using ENFA, and including suitable habitat predicted in all three models.

Table S2 showed that the Western and the *edwardsianus* lineage had the highest overlap in all ensembles and ENFA, followed by the *edwardsianus* and Central lineage. Spatial overlap was smallest for the Central and the Western lineage. For ENFA and ensembles including suitable habitat predicted by two models, overlaps including the Central lineage overlapped on average in 9.4% of the predicted cells, while overlaps between the *edwardsianus* and the Western lineage overlapped in 23.1%.

Table S2. Percentage of spatial niche overlap (*N* overlapping cells*100/sum of suitable cells for both lineages) estimated by different types of ensembles: a) 2 or 3 models, b) 3 models, or c) by ENFA (see also main text)

a)

**MDE10**	Central lineage	Western lineage
edwardsianus	16.04%	21.65%
Central lineage	-	4.45%

**MDE7**		
edwardsianus	16.67%	24.15%
Central lineage	-	7.12%

b)

**MDE10**		
edwardsianus	0.84%	0.96%
Central lineage	-	0%

**MDE7**		
edwardsianus	1.39%	8.16%
Central lineage	-	0.55%

c)

edwardsianus	9.4%	23.4%
Central lineage	-	2.8%

For each type of ensembles we varied the number of predictors and used either the upper three quartiles of the best predicting predictors estimated by ENFA (MDE10) or the first half of predictors (MDE7) for MDE analyses. All ensembles were obtained combining models of ENFA, MaxEnt and MDE.

2. Interpredictivity among MaxEnt models

For each lineage we used one-way ANOVAs to compare habitat suitability predicted by MaxEnt models for the sampled populations (N = 22) among lineages (Figure S2), and thus identical methodology used as for HS scores obtained by ENFA (see main text, Figure 9).

HS scores significantly differed between lineages in models predicting the *edwardsianus* lineage ($F_{2,19}$ = 3.45, P = 0.05, Figure S2a). *Post-hoc* comparisons showed that HS scores of sampled *edwardsianus* populations tended to be higher than those of the other two clades (Tukey range tests: P_{adj} = 0.09 in both cases), and no significant differences were present between the Central and the Western lineage (P_{adj} = 0.95). HS scores were significantly different between lineages ($F_{2,19}$ = 4.35, P = 0.02, Figure S2b) in models derived for the Central lineage (Figure 2Sb). *Post-hoc* comparisons showed that the scores of the Central lineage were higher than for those of the Western lineage (P_{adj} = 0.02) and tended to be higher than those of the *edwardsianus* lineage (P_{adj} = 0.14). No significant differences were present between the Western and the *edwardsianus* lineage (P_{adj} = 0.48). HS scores derived for the Western lineage significantly differed between lineages ($F_{2,19}$ = 10.50, P < 0.01, Figure S2c), and *post-hoc* test showed that HS scores of the Western lineage were significantly higher than those of the other two lineages (P_{adj} < 0.01, in both cases). No significant differences existed between the Central and the *edwardsianus* lineage (P_{adj} = 0.50).

In summary, results from MaxEnt and ENFA predictions were qualitatively similar.

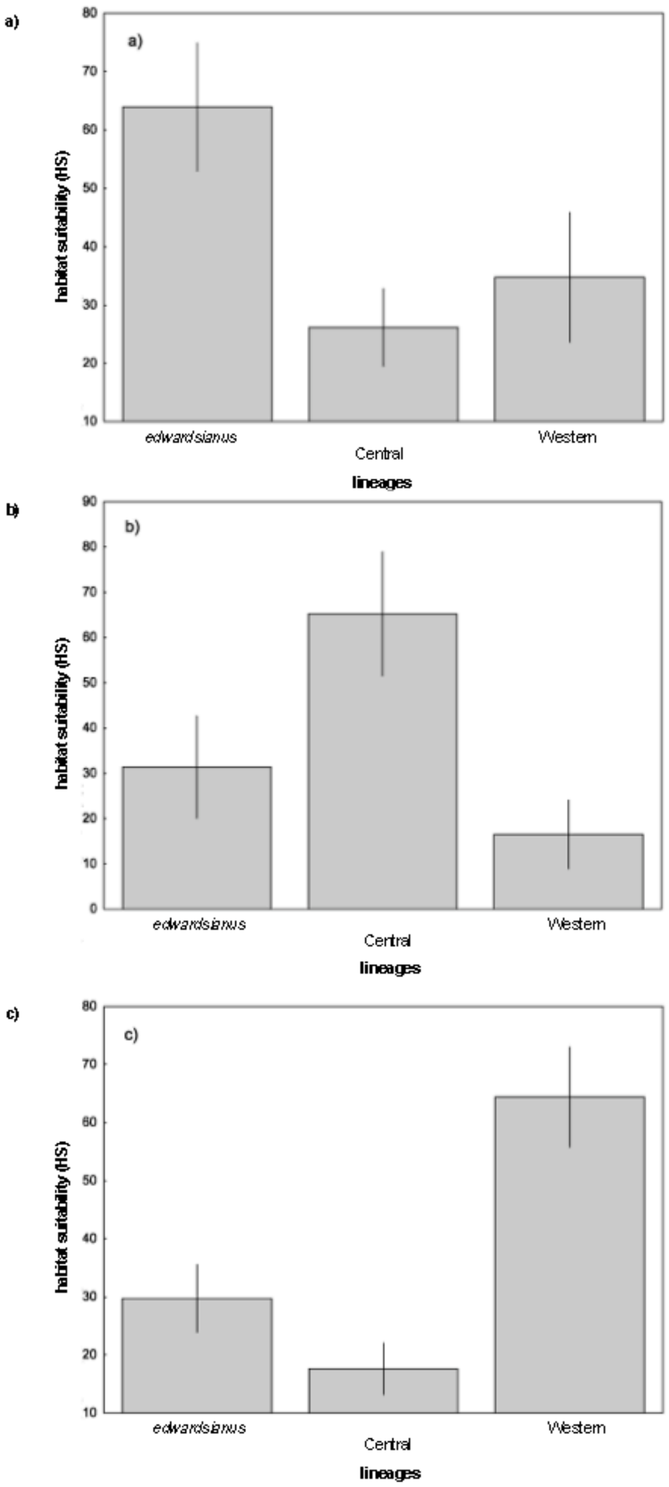

Figure S2. Differences in habitat suitability (of sampled populations) between *P. hispanicus* lineages, predicted by MaxEnt. Average (±SE) HS scores derived from models for a) *edwardsianus* lineage, b) Central lineage, and c) Western lineage are shown.

3. References

1. Phillips SJ, Anderson RP, Schapire RE: **Maximum entropy modelling of species geographic distributions**. *Ecol Model* 2006, **190**:231-259.
2. Busby JR: **BIOCLIM - A bioclimate analysis and prediction system**. In: *Nature Conservation: Cost effective biological surveys and data analysis.* Edited by Margules CR, Austin MP. Melbourne: CSIRO; 1991: 64-68.
3. Jiménez-Valverde A, Peterson AT, Soberón J, Overton JM, Aragón P, Lobo JM: **Use of niche models in invasive species risk assessments**. *Biol Inv* 2011:in press.
4. Jiménez-Valverde A, Lobo JM, Hortal J: **Not as good as they seem: the importance in concepts in species distribution modelling**. *Divers Distrib* 2008, **14**:885-890.
5. Soberón J, Nakamura M: **Niches and distributional areas: concepts, methods, and assumptions**. *Proce Natl Acad USA* 2009, **106**:19644-19650.
6. Aragón P, Baselga A, Lobo JM: **Global estimation of invasion risk zones for the western corn rootworm *Diabrotica virgifera virgifera*: integrating distribution models and physiological thresholds to assess climatic favourability**. *J Appl Ecol* 2010, **47**:1026-1035.
7. Araújo MB, New M: **Ensemble forecasting of species distributions**. *Trend Ecol Evol* 2007, **22**:42-47.
